# A quantitative micro-tomographic gut atlas of the lepidopteran model insect *Manduca sexta*

**DOI:** 10.1016/j.isci.2023.106801

**Published:** 2023-05-05

**Authors:** Anton G. Windfelder, Jessica Steinbart, Ulrich Flögel, Jan Scherberich, Marian Kampschulte, Gabriele A. Krombach, Andreas Vilcinskas

**Affiliations:** 1 Branch Bioresources, Fraunhofer Institute for Molecular Biology and Applied Ecology IME, Giessen, Germany; 2 Laboratory of Experimental Radiology, Justus Liebig University Giessen, Giessen, Germany; 3 Department of Diagnostic and Interventional Radiology, University-Hospital Giessen, Germany; 4 Experimental Cardiovascular Imaging, Molecular Cardiology, Heinrich Heine University, Düsseldorf, Germany; 5 Institute for Insect Biotechnology, Justus Liebig University Giessen, Giessen, Germany

**Keywords:** Imaging anatomy, Methodology in biological sciences, Model organism

## Abstract

The tobacco hornworm is used extensively as a model system for ecotoxicology, immunology and gut physiology. Here, we established a micro-computed tomography approach based on the oral application of the clinical contrast agent iodixanol, allowing for a high-resolution quantitative analysis of the *Manduca sexta* gut. This technique permitted the identification of previously unknown and understudied structures, such as the crop or gastric ceca, and revealed the underlying complexity of the hindgut folding pattern, which is involved in fecal pellet formation. The acquired data enabled the volume rendering of all gut parts, the reliable calculation of their volumes, and the virtual endoscopy of the entire alimentary tract. It can provide information for accurate orientation in histology uses, enable quantitative anatomical phenotyping in three dimensions, and allow the calculation of locally effective midgut concentrations of applied chemicals. This atlas will provide critical insights into the evolution of the alimentary tract in lepidopterans.

## Highlights

• This is a quantitative 3D reference atlas of the digestive tract of *M. sexta*
• Oral application of iodixanol allowed a high-resolution 3D analysis of the gut
• Revealing structures like the crop, gastric ceca or the complex gut folding pattern
• Gut volumes can be used to calculate local concentrations of oral-applied compounds

## Graphical abstract

**Figure:**
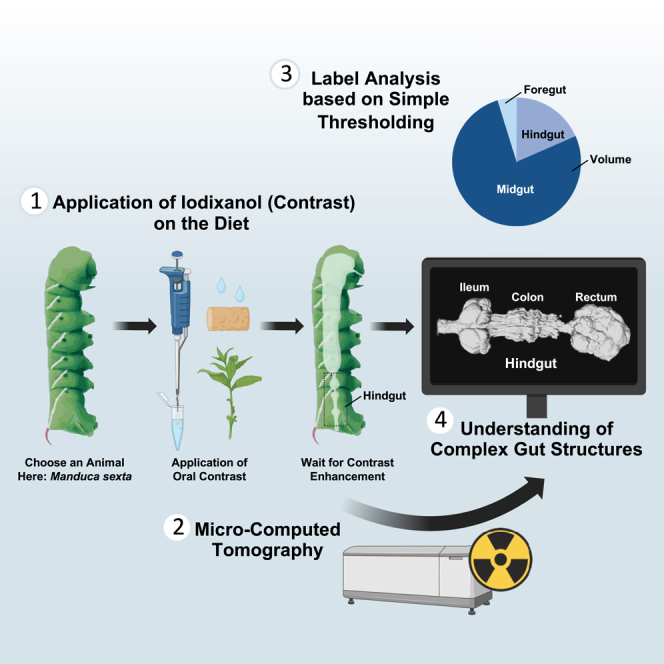

## Introduction

The tobacco hornworm (*Manduca sexta*) is one of the most important lepidopteran model organisms. It can be bred inexpensively in large numbers,^1^^,^^2^^,^^3^ and key benefits include its fully assembled genome,^4^^,^^5^ methylome^6^ the availability of antibodies,^7^ and its large size (>10 g),^8^ making it ideal for studies in biochemistry,^4^ developmental biology,^9^^,^^10^ immunology,^11^ epigenetics,^12^ imaging,^9^^,^^13^ morphology,^9^^,^^14^ neurobiology,^15^^,^^16^ and gut physiology.^17^ Such models are required because lepidopterans are among the most devastating agricultural pests, substantially reducing the global yield of cereal, soybean and potato crops.^18^ Effective species-restricted insecticides against lepidopteran pests are therefore urgently needed.^19^ On the other hand, butterflies and moths provide economically critical pollination services, so it is important to avoid habitat destruction and the extensive use of nonspecific insecticides that reduce insect biodiversity.^20^^,^^21^ In this context, *M. sexta* is used as a model in ecotoxicology and agricultural science to study and develop new insecticides.^12^^,^^16^^,^^19^^,^^22^^,^^23^^,^^24^^,^^25^ Lately, lepidopteran insects were also used as high-throughput preclinical models for diseases involving gut inflammation, including inflammatory bowel disease, which reflects the general homology of the innate immune system and the common epithelial organization of the alimentary tract in insects and mammals.^26^^,^^27^^,^^28^^,^^29^^,^^30^ The high-throughput screening of large compound libraries can help to identify new inhibitors against evolutionarily conserved targets. This strategy can economize on experiments with small mammals such as mice or rats according to the 3R principle (replace, reduce and refine).^31^

Standard hypothesis testing in *M. sexta* often involves histological analysis following the oral application of a specific agent.^32^^,^^33^ Histology allows the analysis of physiological or pathological processes at the cellular level^34^ but is restricted to two dimensions, whereas most anatomical and pathological patterns are three-dimensional.^35^ Furthermore, preparing histological samples is a slow and labor-intensive process, and the fixation and sectioning steps introduce tissue distortion and shrinkage artifacts.^36^^,^^37^^,^^38^ Accordingly, researchers tend to focus on a tiny fraction of the sample for microscopic analysis, which leads to a significant loss of information.^35^

Several 3D imaging modalities are suitable for anatomical analysis, including micro-magnetic resonance imaging (μMRI),^39^ synchrotron X-ray imaging,^40^^,^^41^ and computed tomography (CT).^30^ The resolution of images generated by μMRI is too low for detailed anatomical studies, even in large insects like *M. sexta*,^9^ whereas synchrotron X-ray imaging or histotomography offers high-resolution images comparable to microscopy but the field of view in most synchrotron facilities is limited to a few millimeters, making it impractical for large insects.^35^ Furthermore, the procedure is very expensive and limited in availability.^35^ CT modalities close the gap between synchrotron X-ray imaging and μMRI, but the field of view is too restricted in nano-CT, which leaves benchtop micro-CT (μCT) as the only practical option for the high-resolution 3D imaging of *M. sexta*.^35^ Benchtop μCT is an emerging imaging modality used in recent years as a comprehensive tool for studying coleopteran,^42^^,^^43^^,^^44^^,^^45^^,^^46^^,^^47^ dipteran,^48^^,^^49^^,^^50^^,^^51^^,^^52^^,^^53^ hymenopteran,^54^^,^^55^^,^^56^^,^^57^ and hemipteran^44^^,^^58^^,^^59^ anatomy. It is also used to study the anatomy of spiders,^60^ amphipods,^61^ decapods,^62^ annelids,^63^^,^^64^ snails,^65^ squids,^66^ and cnidarians.^67^

Here, we used μCT to generate a comprehensive 3D anatomic atlas of the larval *M. sexta* alimentary tract. The oral application of the clinical contrast agent iodixanol was combined with a hydrated scanning method, which is faster and more accurate than the traditional dry scanning protocols for insects and can resolve the morphology of the alimentary tract in unprecedented detail.^42^^,^^44^^,^^58^^,^^68^ Importantly, this method is compatible with fresh, unfixed specimens to generate accurate, quantitative data without tissue shrinkage or swelling artifacts caused by standard fixation solutions.^36^^,^^37^^,^^38^^,^^69^^,^^70^ This allowed the precise segmentation of the alimentary tract based on trash-holding, a rapid segmentation method that does not require extensive computational resources. Our findings are animated in Videos S1, S2, S3, S4, S5, S6, S7, and S8, which can be used as teaching materials in schools and universities.

**Figure 1:**
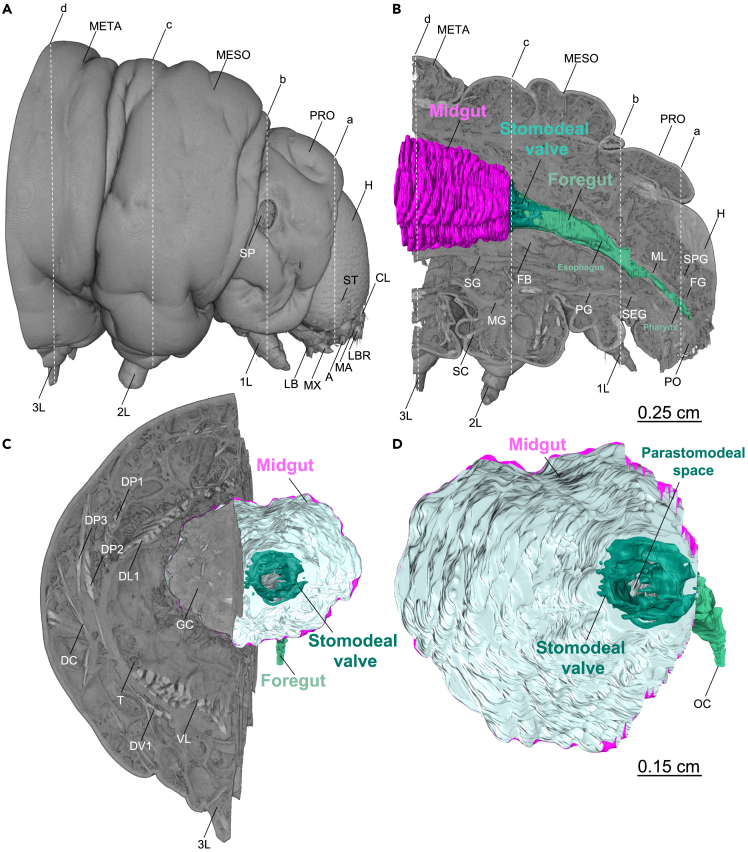
Volume rendering of the thorax and head of a late L5d6 *Manduca sexta* larva (iodine-contrasted dry scan, whole-mount) (A) Volume rendering of the thorax and head (lateral view). 1L–3L: first to third leg, A: antenna, CL: clypeus, H: head, LB: labium, LBR: labrum, MA: mandible, MESO: mesothorax, META: metathorax, MX: maxilla, PRO: prothorax, SP: spiraculum, ST: stemma (simple eye). (B) Volume rendering of the thorax and head (sagittal cross-section, lateral view) with segmented foregut (pharynx and esophagus), stomodeal valve, and the anterior midgut. 1L–3L: first to third leg, FB: fat body, FG: frontal ganglion, H: head, MESO: mesothorax, META: metathorax, MG: mesothoracic ganglion, ML: muscular layer of the foregut, PG: prothoracic ganglion, PRO: prothorax, PO: preoral cavity with mouth parts, SC: spiracular muscle, SEG: subesophageal ganglion, SG: silk glands, SPG: supraesophageal ganglion or brain. (C) Volume rendering of the thorax and head (sagittal and axial cross-section, caudal view), segmented foregut, stomodeal valve, and the anterior midgut. 3L: third leg, DC: dorsocoxal muscle, DL: dorsolongitudinal muscle, DP: dorsopleural muscle, DV: dorsoventral muscle, GC: gut content, T: trachea, VL: ventrolongitudinal muscle. (D) Segmented foregut, stomodeal valve, and the anterior midgut (sagittal cross-section, caudolateral view). OC: oral cavity (functional mouth). The lines labeled (A, B, C, and D) indicate the frontal planes shown in Figure 2. An animated version of this volume rendering with a virtual endoscopy of the stomodeal valve and foregut is provided in Video S1. Table S1 summarizes the abbreviations and terminology of the anatomical structures.

**Figure 2:**
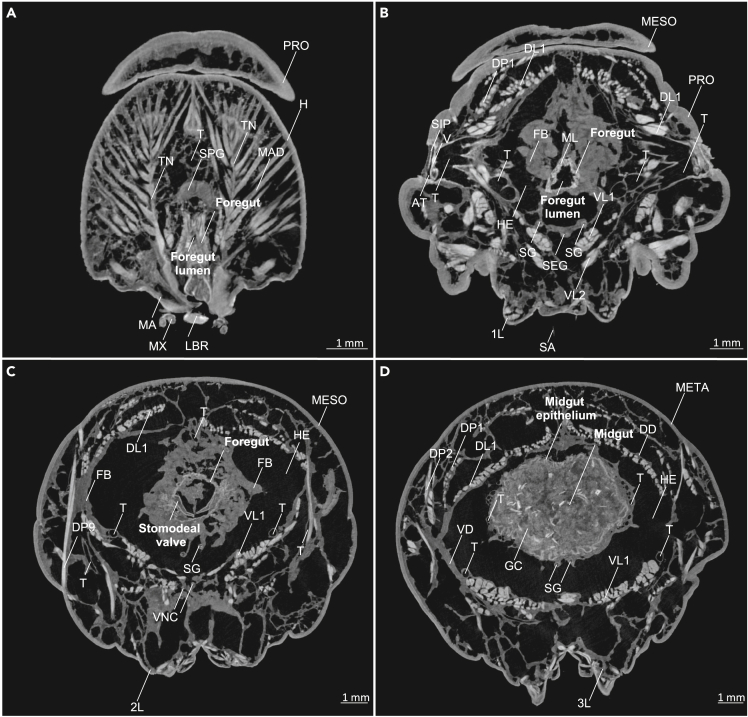
Axial μCT images (frontal plane) of the thorax and head of a late L5d6 *Manduca sexta* larva (iodine-contrasted dry scan, whole-mount) (A) Axial cross-section of the head with a cross-section of the supraesophageal ganglion or brain (SPG). PRO: prothorax (first thoracic segment), H: head, LBR: labrum, MA: mandible, MAD: mandibular adductor, MX: maxilla, T: trachea, TN: tentorium. (B) Axial cross-section of the mesothorax with a cross-section of the foregut. 1L: first leg, AT: atrium, DL: dorsolongitudinal muscle, DP: dorsopleural muscle, FB: fat body, HE: hemocoel, MESO: mesothorax (second thoracic segment), ML: muscular layer of the foregut, PRO: prothorax (first thoracic segment), SA: seta, SEG: subesophageal ganglion, SG: silk gland, SIP: sieve plate, T: trachea, V: valve, VL: ventrolongitudinal muscle. (C) Axial cross-section of the mesothorax with a cross-section of the stomodeal valve. 2L: second leg, DL: dorsolongitudinal muscle, DP: dorsopleural muscle, FB: fat body, HE: hemocoel, MESO: mesothorax (second thoracic segment), SG: silk gland, T: trachea, VL: ventrolongitudinal muscle, VNC: ventral nerve cord. (D) Axial cross-section of the metathorax with a cross-section of the midgut. 3L: third leg, DD: dorsal diaphragm, DL: dorsolongitudinal muscle, DP: dorsopleural muscle, GC: gut content, HE: hemocoel, META: metathorax (third thoracic segment), SG: silk gland, T: trachea, VD: ventral diaphragm, VL: ventrolongitudinal muscle. An animated version of these μCT scans with a virtual endoscopy of the stomodeal valve and foregut is provided in Video S1. Table S1 summarizes the abbreviations and terminology of the anatomical structures.

Video S1. Volume rendering of a late L5d6 *Manduca sexta* larva with virtual endoscopy of the stomodeal valve and foregut, related to Figures 1 and 2

Video S2. Volume rendering of an early L5d2 *Manduca sexta* larva with an introduction to the general anatomy of the alimentary tract, related to Figure 5

**Figure 3:**
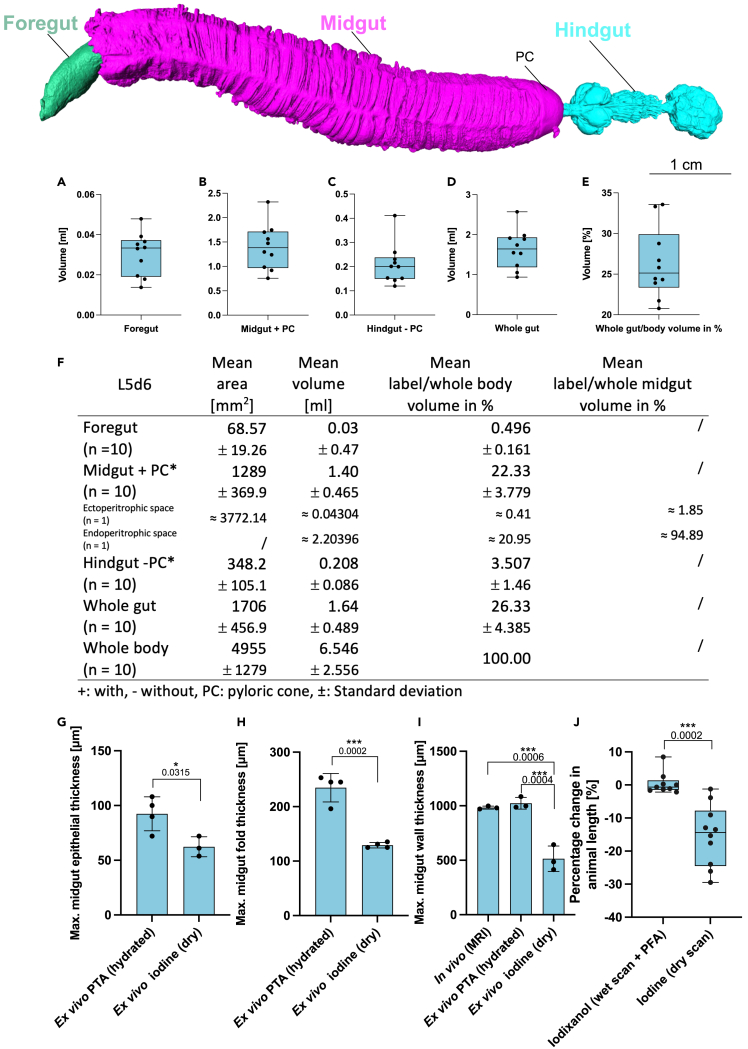
Mean area, volume or thickness of different *Manduca sexta* L5d6 gut parts (A–F) Mean (n = 10) area, mean volume and proportional volume based on iodixanol-contrasted hydrated scans of whole-mount larvae. (G–I) Mean thickness based on PTA-contrasted hydrated scan and iodine-contrasted dry scan of isolated gut parts or *in vivo* μMRI. (G) Maximum midgut epithelial thickness (unpaired *t* test). (H) Maximum midgut fold thickness (unpaired *t* test). (I) Maximum midgut wall thickness (one-way ANOVA, F(2,6) = 42.87, R2 = 0.9346, p = 0.0003). (J) shows the tissue shrinkage of the introduced oral iodixanol method when used with PFA fixation to the traditional iodine dry scan protocol (Mann-Whitney test). The following significance levels have been used: ns = p>0.05, ∗ = p≤0.05, ∗∗p≤0.01, ∗∗∗ = p≤0.001 and ∗∗∗∗ = p≤0.0001. Boxplots: 25th–75th percentiles, whiskers: min-max (show all points), center: median). Bar charts represent mean and SD. Every data point represents a single animal. ∗Note that the pyloric cone (PC) is a part of the hindgut not the midgut, but because these parts share the same luminal cavity, the midgut volume and area calculations included the pyloric cone (which has the same color code as the midgut).

Video S3. Volume rendering of a late L5d6 *Manduca sexta* larva with virtual endoscopy of the alimentary tract, related to Figures 3,7, and 8

**Figure 4:**
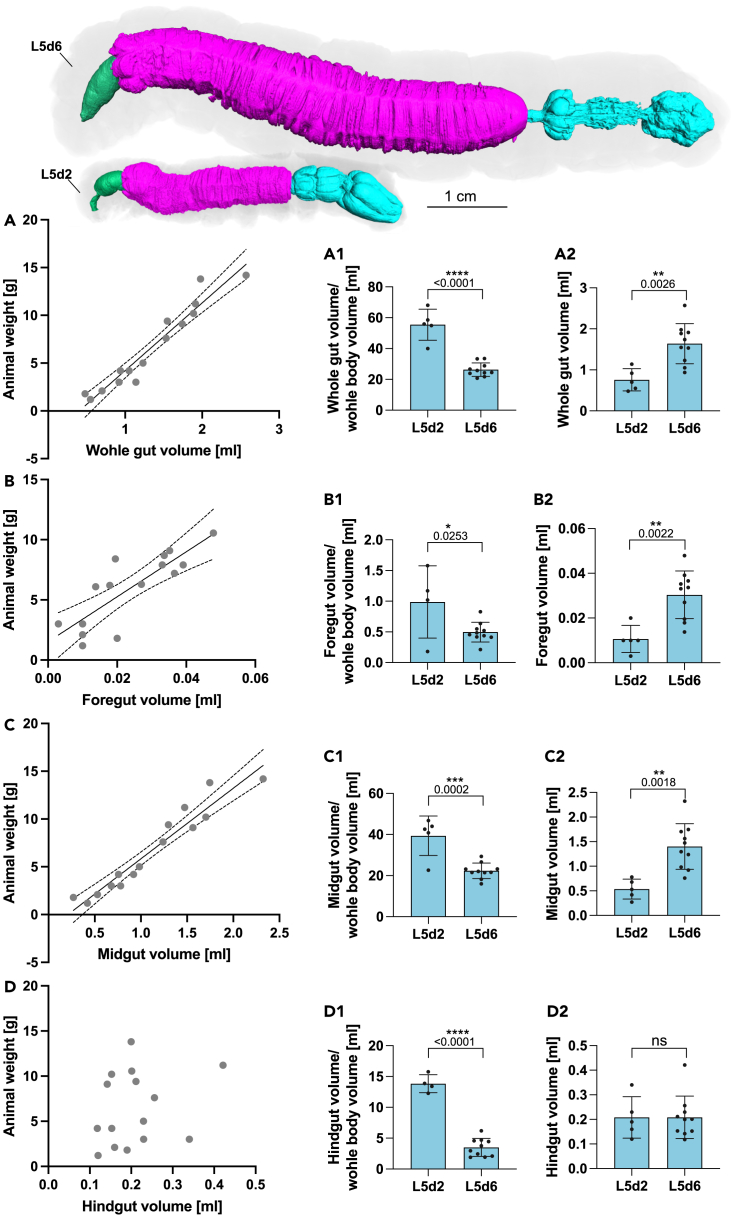
Comparison of different gut parts with animal weight and relative and absolute volume (oral iodixanol contrasting, hydrated scan, whole-mount) Comparison of early L5d2 (n = 5) and late L5d5 (n = 10) larvae (with Pearson correlation, simple linear regression and unpaired t tests). **A** shows the whole gut volume in relation to the weight of L5d2 and L5d5 animals: R^2^ = 0.9313, F(1,13) = 176, P = <0.0001 and r = 0.965, P = <0.0001. **A1** shows a relative and **A2** absolute comparison of the whole gut volume in early L5d2 and late L5d5 larvae. **B** shows the foregut volume in relation to the weight of L5d2 and L5d5 animals: R^2^ = 0.6840, F(1,13) = 28.14, P = 0.0001 and r = 0.827, P = <0.0001. **B1** shows a relative and **B2** absolute comparison of the foregut volume in early L5d2 and late L5d5 larvae. **C** shows the midgut volume in relation to the weight of L5d2 and L5d5 animals: R^2^ = 0.9255, F(1,13) = 161.5, P = <0.0001 and r = 0.962, P = <0.0001. **C1** shows a relative and **C2** absolute comparison of the midgut volume in early L5d2 and late L5d5 larvae. **D** shows the hindgut volume in relation to the weight of L5d2 and L5d5 animals: R^2^ = 0.05785, F(1,13) = 0.7982, P = ns and r = 0.2405, P = ns. **D1** shows a relative and **D2** absolute comparison of the hindgut volume in early L5d2 and late L5d5 larvae. The following significance levels have been used: ns = P > 0.05, ∗ = P ≤ 0.05, ∗∗ P ≤ 0.01, ∗∗∗ = P ≤ 0.001 and ∗∗∗∗ = P ≤ 0.0001. Bar charts represent mean and SD. Every data point represents a single animal. Scatter plots show the 95 CI (dashed lines) and the trend line (lines).

Video S4. Segmentation of the alimentary tract in *Manduca sexta* larvae at three developmental stages: early first instar (L1d1), early fifth instar (L5d2), and late fifth instar (L5d6), related to Figures 4 and 8

Video S5. Segmentation of the endoperitrophic space of a late L5d6 *Manduca sexta* larva, related to Figure 9

Video S6. Volume rendering of the hindgut of a late L5d6 *Manduca sexta* larva, related to Figure 10

Video S7. Hindgut morphology and fecal pellet formation in an early L5d2 *Manduca sexta* larva, related to Figure 11

Video S8. Volume rendering of a fecal pellet from a late L5d6 *Manduca sexta* larva, related to Figure 11

## Results

### Strategy for the development of a 3D anatomic atlas

The 3D anatomic atlas was designed to provide anatomical details with particular emphasis on the gut from the egg to the last larval stage of *Manduca setxa* (Figures S1–S4, 1, and 2) and quantitative volumetric data representing the larval stages used in most screening experiments,^7^^,^^25^^,^^71^ specifically the first instar on developmental day 1 (L1d1, n = 8), the fifth instar on developmental day 2 (L5d2, n = 7), and the fifth instar on developmental day 6 (L5d6, n = 37). In total, 54 micro-CT scans using 49 animals have been used in this paper. Table S1 summarizes the anatomical abbreviations used in this article to help with the understanding of labeled images and videos.

### The biting and chewing mouthparts

The biting and chewing mouthparts of *M. sexta* larvae (Figures 1A, 1B, S5 and Video S1) facilitate the ingestion of food. The labrum (LBR; Figures 1A and S5) is a plate that arises from the clypeus (CL) and functions as the upper lip. The paired mandibles (MA; Figures 1A and S5) shred and cut food. Together with the prominent mandible adductor (MAD; Figure 2A and Video S1), which dominates the head capsule, they form the most prominent part of the chewing apparatus. The paired maxillae (MX; Figures 1 and S5) are used to manipulate and probe food. Finally, the labium (LB; Figures 1A and S5) with the mouthparts discussed above encloses the preoral cavity (PO; Figure 1B, and Video S1).

### General organization of the alimentary tract

The alimentary tract of *M. sexta* larva and most insects can be divided into three regions: the cuticle-lined foregut (stomodeum), the midgut (mesenteron), and the cuticle-lined hindgut (proctodeum).^72^ These are discussed in turn below, and are highlighted in Video S2.

### The foregut

The larval foregut of *M. sexta* begins with the oral cavity or mouth (OC; Figures 1B, 1D and Video S1) adjacent to the proximal side of the preoral cavity (PO; Figure S6). The oral cavity is followed by the pharynx (Figures 1B and S6), which features a prominent muscular layer (ML; Figures 1B, 2B, and Video S1) suggesting a fundamental function in ingestion. Next, the pharynx follows a path ventral relative to the frontal ganglion (FG) and supraesophageal ganglion (brain, SPG; Figures 1B, 2A, and Video S1). The pharynx then passes the subesophageal ganglion dorsally (SEG; Figure 1B) and joins the esophagus (Figure 1). Of interest, the esophagus is significantly less muscular than the pharynx and seems highly expandable (crop, see below, Figures 1B and S7 and Video S1). Finally, at the level of the mesothorax (MESO; Figures 1B, 2C, S6A, and S6B and Video S1), the foregut joins the midgut. This transition is the position of the stomodeal valve (valvula cardiaca or cardiac valve; Figures 1, 2C, S6, and S8, and Video S1), which projects into the midgut lumen (Figures S6 and S8, and Video S1). The stomodeal valve impedes the retrograde movement of food from the midgut to the foregut and may thus serve to regulate regurgitation.^73^^,^^74^ In caterpillars, regurgitation is an important defensive strategy against predators.^75^ Figure S6 shows the stomodeal valve from distal and proximal perspectives (Figures S6D and S6E). Finally, our μCT analysis revealed that the *M. sexta* larval foregut can develop a crop. All L5d2 but not the other studied stages had a crop at the level of the esophagus, which implies that this foregut region is quite flexible (Figures S7, 7G, and 7H).

The mean volume of the L1d1 foregut was 0.0014 μL with a mean area of 0.124 mm^2^ (Tables S2A and S2F). The mean volume of the L5d2 foregut was 0.0106 mL with a mean area of 36.8 mm^2^ (Table S3A and S3F). The mean volume of the L5d6 foregut was 0.03 mL with a mean area of 68.57 mm^2^ (Figures 3A and 3F). The volume of the foregut as a proportion of the total larval volume was 0.07% for L1d1 (Table S2F), 0.1% for L5d2 (Table S3F) and 0.5% for L5d6 (Figure 3F). The foregut volume of L5d6 animals was significantly higher than in L5d2 animals and positively correlated with animal weight. Accordingly, the foregut volume could be predicted based on animal weight (Figures 4B–B2).

### The midgut

**Figure 5:**
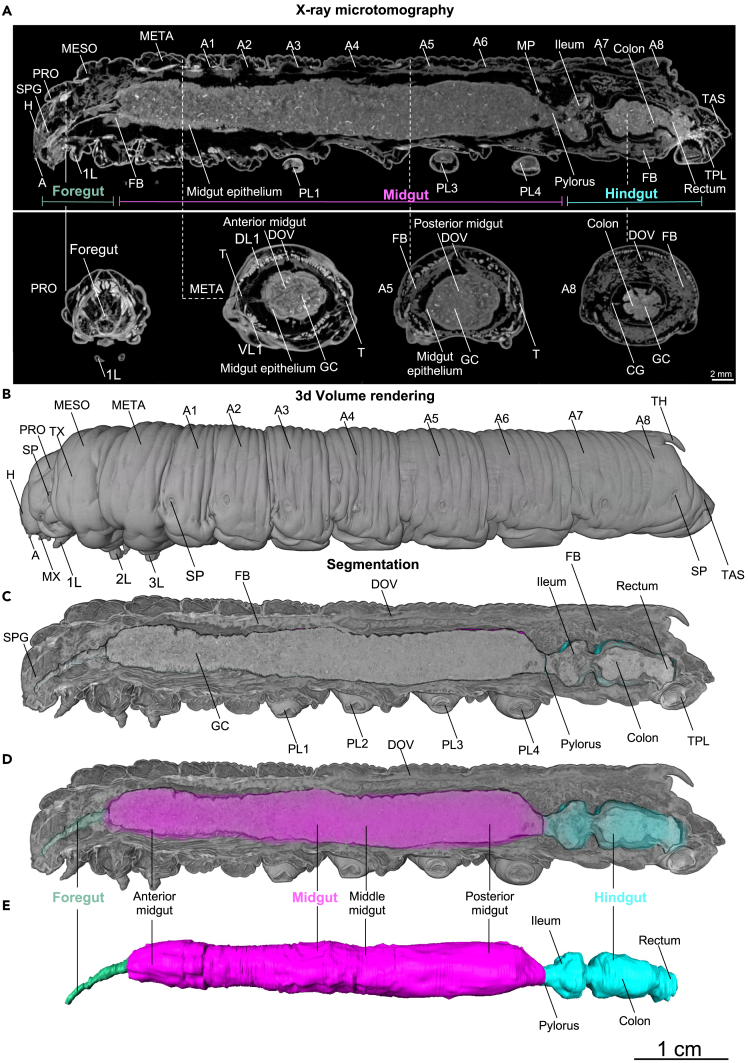
Volume renderings and segmentation of the alimentary tract of a late L5d6 *Manduca sexta* larva (iodine-contrasted dry scan, whole-mount) based on μCT images (A) Sagittal plane of an early L5d6 larva with corresponding axial images (foregut, anterior midgut, posterior midgut, and hindgut). 1L: leg 1, A: antenna, A1–A8: abdominal segments 1–8, TAS: terminal abdominal segment, CG: coleo-groove, DL: dorsolongitudinal muscle, DOV: dorsal vessel, FB: fat body, GC: gut content, H: head, MESO: mesothorax (second thoracic segment), META: metathorax (third thoracic segment), MP: malpighian tubules, SPG: supraesophageal ganglion or brain, PL1–4: prolegs 1–4, PRO: prothorax (first thoracic segment), T: trachea, TPL: terminal proleg, VL: ventrolongitudinal muscle. (B) Volume rendering corresponding to the μCT images shown in (A). 1–3L: legs 1–3, A: antenna, A1–A8: abdominal segments 1–8, H: head, MESO: mesothorax (second thoracic segment), META: metathorax (third thoracic segment), MX: maxilla, PRO: prothorax (first thoracic segment), SP: spiraculum, TAS: terminal abdominal segment, TH: terminal horn, TX: thorax. (C and D) Sagittal cross-section of the volume rendering shown in (B), with segmented alimentary tract. DOV: dorsal vessel, FB: fat body, GC: gut content, PL1–4: prolegs 1–4, SPG: supraesophageal ganglion or brain, TPL: terminal proleg. (E) Isolated segmented alimentary tract shown in (D). An animated version of this volume rendering is provided in Video S2. Table S1 summarizes the abbreviations and terminology of the anatomical structures.

**Figure 6:**
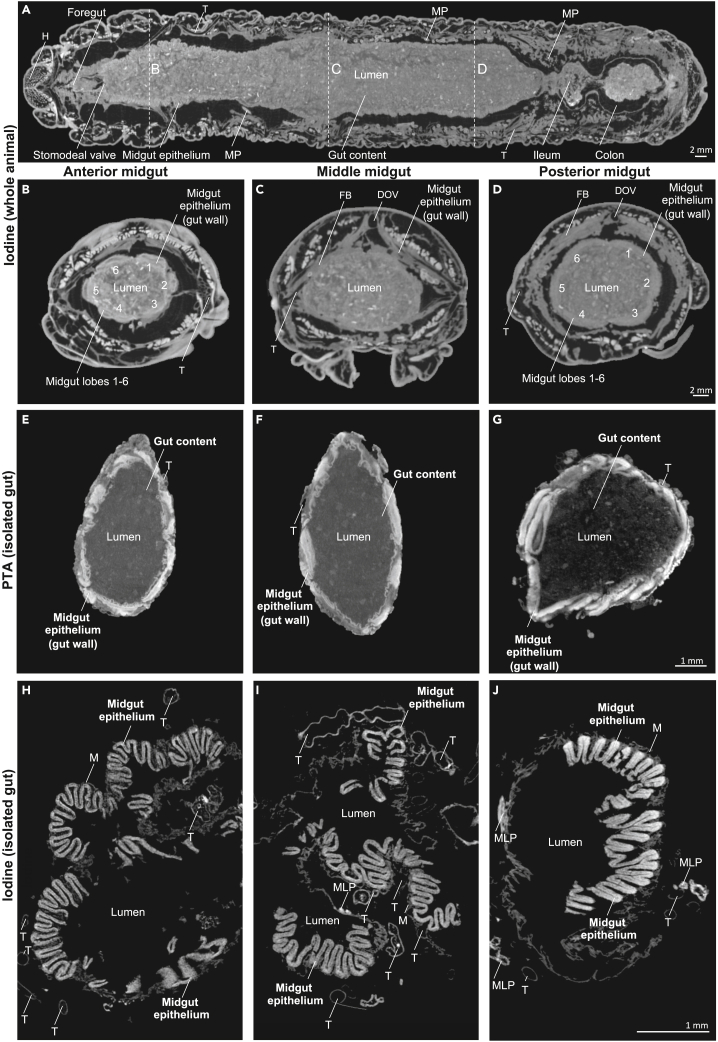
The alimentary system of a late L5d6 *Manduca sexta*larva based on μCT images with different contrast and preparation methods (A–D) Whole *M. sexta* larvae contrasted with 1% iodine followed by chemical dehydration and air drying (A coronal, B-D axial orientations). (E–G) Isolated midgut, axial orientation, stained with 1% PTA (no drying). (H–J) Isolated midgut, axial orientation, stained with 1% iodine (critical point drying). DOV: dorsal vessel, FB: fat body, H: head, M: visceral muscle (gut), MP: Malpighian tubules, T: trachea. The numbers indicate the different midgut lobes (1–6). Table S1 summarizes the abbreviations and terminology of the anatomical structures.

The midgut is the largest organ of the *M. sexta* larva and extends from the mesothorax to the sixth abdominal segment (Figures 5A, 5C, and Video S2). It is subdivided into anterior, middle and posterior compartments (Video S2).^76^ The anterior midgut epithelium is heavily folded compared to the other two parts (Figures 5A, 5C, 6A, 6B, 6E, 6H, and S8–S12 and forms six distinct midgut lobes when viewed along the axial (or transversal) plane (Figures 6B–6D).

The anterior, middle and posterior midguts were dissected, contrasted with iodine, and prepared by critical point drying, revealing the cellular epithelium in a high spatial resolution suitable for quantitative histological phenotyping of the gut epithelium (Figures 6H–6J). However, the epithelial folding pattern was only revealed in full when contrasted with phosphotungstic acid (PTA) followed by hydrated scans or in whole-mount iodine-contrasted dry scans. Transversal folding dominated the epithelial folding of the midgut (Figures 6A, S9A–S9C, and S10). In addition, the transversal folding of the midgut involved strong undulations in the anterior and posterior midgut (Figures S9A and S9B). The epithelial folding pattern was most evident on the sagittal planes (Figures 6A, S8, S9A, S9B, and S10). It showed second-order folding in the anterior and posterior midgut and single-order folding in the middle midgut (Figures S9C and S10).

Of interest, when the gut was contrasted with PTA and scanned without drying, the epithelial monolayer could be seen from the axial perspective as a stratified gut wall because multiple gut folds were cross-sectioned (Figures 6E–6G). In this context, it becomes clear that the axial thickness of the midgut wall almost exclusively reflects the epithelial folding, whereas muscles and tracheae make a negligible contribution (Figures 6E–6G). This differs from the foregut (pharynx Figure 1B) and hindgut pylorus and colon (see next section), which have thick muscular layers in addition to the epithelium.

**Figure 7:**
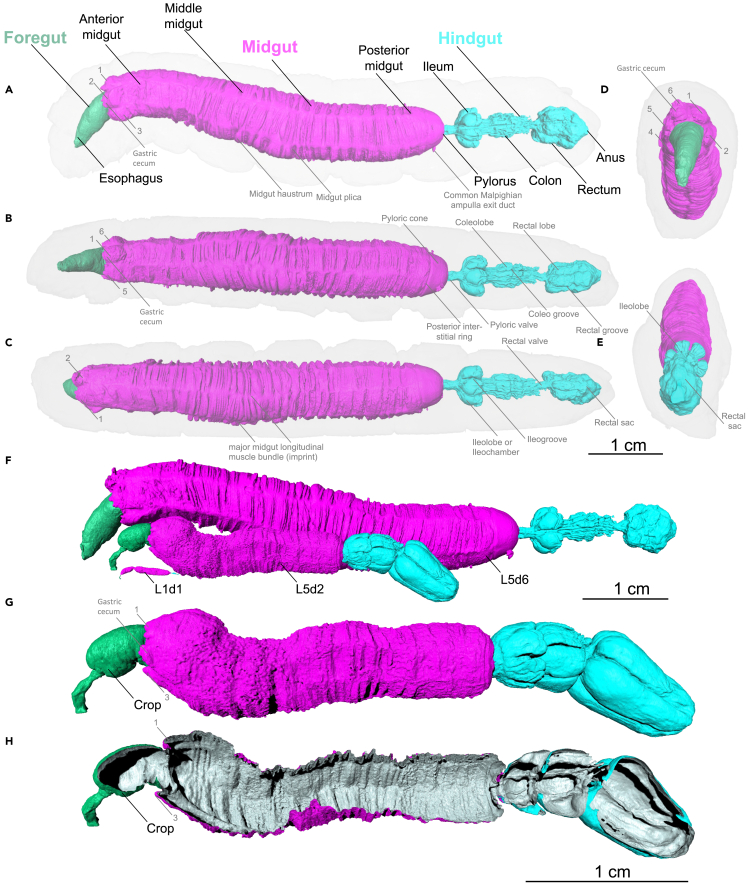
Segmentation of the alimentary tract of a late (L5d6) and an early (L5d2) *Manduca sexta* larva (oral iodixanol contrasting, hydrated scan, whole-mount) The alimentary tract of a late L5d6 larva viewed from the (A) lateral, (B) ventral, (C) dorsal, (D) anterior and (E) posterior orientations. Numbers 1–6 indicate the rudimentary gastric ceca. (F) Comparison of the alimentary tracts of L5d6, L5d2 and L1d1 larvae. An animated version of this volume rendering with a virtual endoscopy of the alimentary tract is provided in Videos S3 and S4. (G and H) show the alimentary tract of a late L5d2 larva viewed from the lateral side with a crop. Note that the pyloric cone (PC) is a part of the hindgut not the midgut, but because these parts share the same luminal cavity, the midgut volume and area calculations included the pyloric cone (which has the same color code as the midgut).

**Figure 8:**
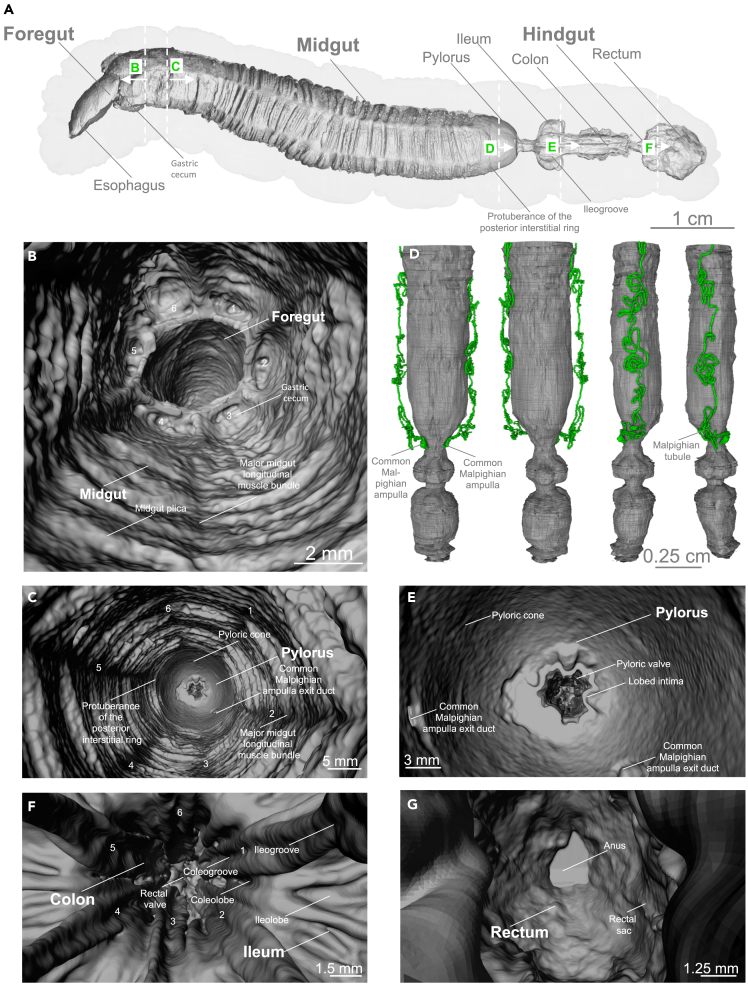
Virtual endoscopy of the alimentary tract of a late L5d6 *Manduca sexta* larva (oral iodixanol contrasting, hydrated scan, whole-mount) (A) Sagittal cross-section of the alimentary tract. The labels B–F indicate the orientation of the following images. (B) Virtual endoscopic view from the anterior midgut to the foregut. Numbers 1–6 indicate the rudimentary gastric ceca. (C) Virtual endoscopic view from the anterior midgut to the posterior midgut. Numbers 1–6 indicate the major midgut longitudinal muscle bundles. (D) Volume rendering of the alimentary tract and the Malpighian tubules (in green) with the two common Malpighian ampullae. (E) Virtual endoscopic view from the posterior midgut to the pylorus, showing the insertions of the two common Malpighian ampullae in the pyloric cone of the midgut. (F) Virtual endoscopic view from the ileum to the colon. Numbers 1–6 indicate the six major ileogrooves. (G) Virtual endoscopic view from the colon to the rectal sac. An animated version of this volume rendering with a virtual endoscopy of the alimentary tract is provided in Video S3.

In an independent experiment, we orally contrasted the alimentary tract with iodixanol (oral iodixanol hydrated scan, whole-mount), which can be seen as a virtual cast preparation (Figures 7, 8, and Video S3). Here, the midgut lumen adopted a hexagonal profile constrained from the six major midgut longitudinal muscle bundles (Figures 6, 7, and Video S3). Protrusions of the gut epithelium covered the hexagonal configuration of the midgut as midgut haustra and plicae (Figures 7, 8 and Video S3). Moreover, oral iodixanol contrasting revealed six prominent protrusions in the most anterior part of the midgut (Figures 7A–7C, 8A, and 8B), the location of gastric ceca in other insects.^73^ Video S4 shows the alimentary tract of L5d6 specimens contrasted with iodixanol in comparison to an L5d2 and a L1d1 specimens. In addition to oral iodixanol, we applied negative, indirect staining of the gut by injecting diatrizoate into the hemocoel (Figure S13). However, the oral application of iodixanol achieved superior contrasting of the alimentary tract.

**Figure 9:**
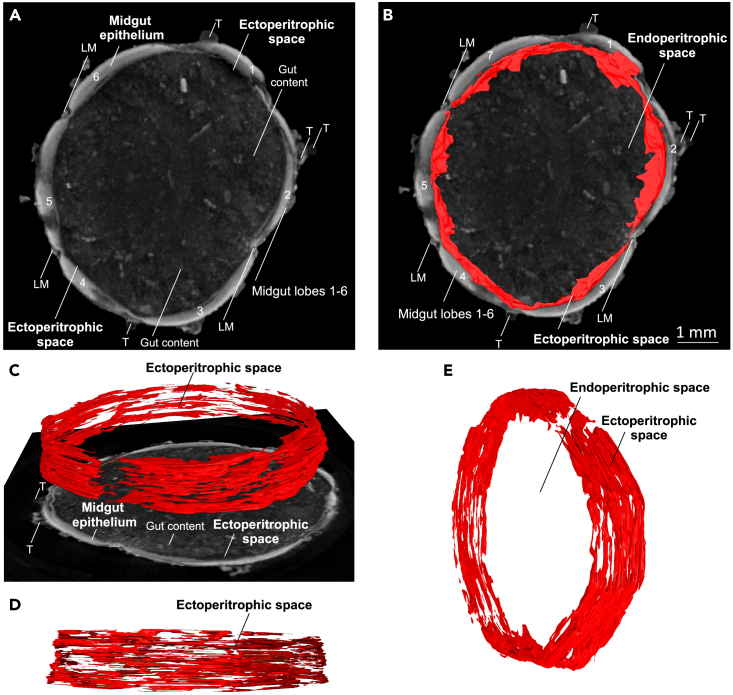
Endoperitrophic and ectoperitrophic spaces of a late L5d6 *Manduca sexta* larva (PTA-contrasted hydrated scan, isolated gut) (A) Isolated midgut from a late L5d6 larva, stained with 1% PTA. (B–E) Segmentation of the endoperitrophic space (red). LM: longitudinal visceral musculature (gut), T: trachea. The numbers indicate the different midgut lobes (1–6). An animated version of this volume rendering is provided in Video S5.

In insects, the midgut is subdivided through the peritrophic matrix into endoperitrophic and ectoperitrophic spaces (Figure 9 and Video S5).^72^ The endoperitrophic space contains the food bolus, whereas the ectoperitrophic space is the area between the outer peritrophic matrix and the midgut epithelium (Figure 9 and Video S5). The endoperitrophic and ectoperitrophic spaces in the L5d6 specimens studied had estimated volumes of 2.20396 mL and 0.04304 mL, respectively (Figure 3F). The ectoperitrophic space had an estimated surface area of 3772.14 mm^2^ (Figure 3F).

Intriguingly, the contrast and scanning procedure significantly influenced the apparent thickness of the midgut epithelium and its folds (Figures 3G–3I). The PTA *ex vivo* hydrated scan yielded significantly thicker specimens than the iodine *ex vivo* dry scan (Figures 3G–3I). Native *in vivo* μMRI measurements of the maximum midgut wall thickness confirmed that the PTA *ex vivo* hydrated scans were close to the *in vivo* situation, which was not the case for the iodine *ex vivo* dry scans (Figure 3I). We also compared the whole mount shrinkage that both methods could introduce. Here the Iodine dry scanning procedure introduced significantly more tissue shrinkage than the iodixanol wet scanning procedure when used with PFA fixation (Figure 3J). Note that the iodixanol wet scanning procedure can also be used without fixation, completely avoiding tissue shrinkage.

The mean volume of the L1d1 midgut was 0.72 μL with a mean area of 7.466 mm^2^ (Tables S2B and S2F). The mean volume of the L5d2 midgut was 0.536 mL with a mean area of 722.8 mm^2^ (Tables S3B and S3F). The mean volume of the L5d6 midgut was 1.4 mL with a mean area of 1289 mm^2^. The volume of the midgut as a proportion of the total larval volume was 34% for L1d1 (Table S2F), 39% for L5d2 (Table S3F), and 22% for L5d6 (Figure 3F). The midgut volume of L5d6 animals was significantly higher than in L5d2 animals and positively correlated with animal weight. Accordingly, the midgut volume could be predicted based on animal weight (Figures 4C–4C2).

### The hindgut

**Figure 10:**
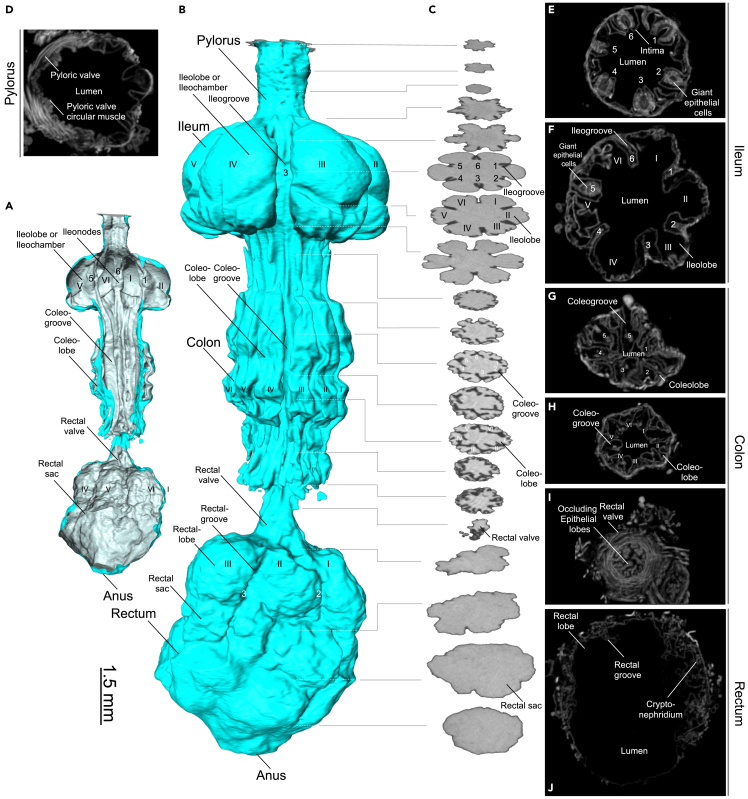
Segmentation of the hindgut in a late L5d6 *Manduca sexta* larva (A and B) Oral iodixanol contrasting, hydrated scan, whole-mount. Lateral view of the hindgut. Roman numerals indicate lobes (I–VI), and numbers indicate nodes (1–6). (C) Axial μCT images of the corresponding volume rendering. (D–J) Iodine-contrasted dry scan, isolated hindgut (axial orientation). An animated version of this volume rendering is provided in Video S6.

**Figure 11:**
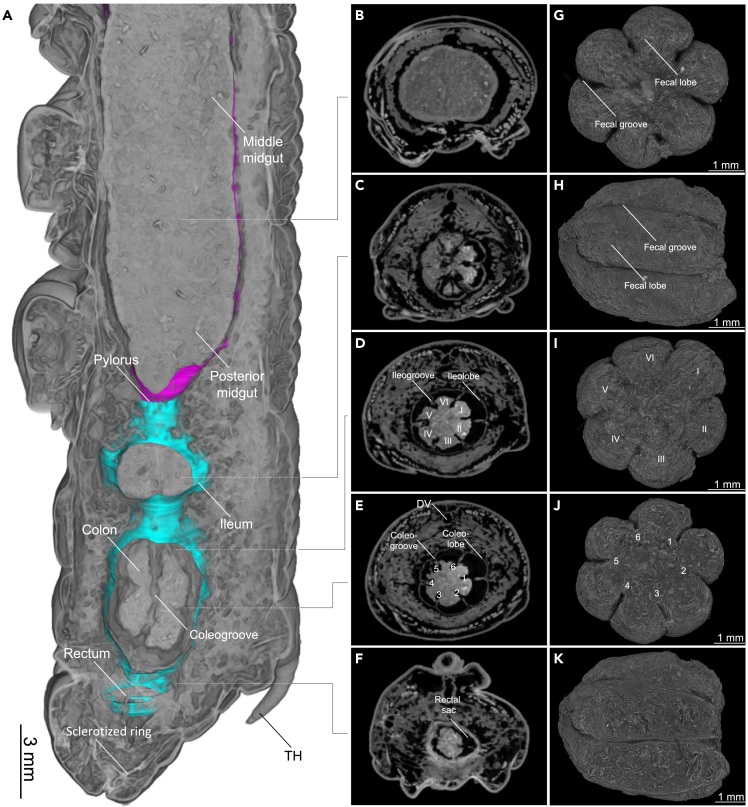
Hindgut morphology and fecal pellet formation in a late L5d6 *Manduca sexta* larva (A–F) Iodine-contrasted dry scan, whole-mount. (A) Sagittal view of the abdominal anatomy, focusing on the posterior midgut and hindgut. (B–F) Axially-orientated μCT images of the same region. (G–K) Volume rendering of a fecal pellet (native). Roman numerals indicate lobes (I–VI), and numbers indicate nodes (1–6). An animated version of this volume rendering is provides in Videos S7 and S8.

The hindgut of most insects can be subdivided into three parts: the pylorus, the ileo-colon, and the rectum.^72^ In *M. sexta* and most other larval macrolepidopertan moths (including bombycoids), the ileo-colon is further subdivided into a separate ileum and colon (Figure 10, 11 and Video S6).^72^

The pyloric cone is the most anterior part of the hindgut. It features an almost smooth luminal surface with two distinct bulges (Figures 7B, 8C, 8E, and S14, and Video S3). Here, the two common Malpighian ampullae (connecting the excretory Malpighian tubules to the hindgut) enter the gut lumen (Figures 8C, 8E, and S14 and Video S3). Although the pyloric cone is a part of the hindgut and not the midgut, these parts share the same luminal cavity. Accordingly, our midgut volume and area calculations included the pyloric cone (Figure 3 and Tables S2 and S3) and the pyloric cone shares the same color code as the midgut in all figures. The next part of the pylorus is the pyloric valve or valvula pylorica (Figure 10D). Here, a strong sphincter muscle (Figure 10D) regulates the passage of materials into more distal parts of the hindgut.

After the confined lumen of the pylorus, the hindgut lumen opens into six discrete ileochambers or ileolobes of the ileum (Figure 10 and Video S6). Here, six non-connected layers of giant epithelial cells synthesize the cuticle intima of the hindgut (Figure S15). Fecal pellet formation starts within the ileum.^14^ The hexagonal folding pattern of the midgut continues through the ileum, the colon, and reaches the rectum (Figures 10 and 11). The colon is a highly inflatable part of the hindgut, shown in its deflated state in Figure 10 and in its inflated state in Figure 11. The rectum (Figure 10) is the principal site of water resorption.^72^ Here, the hindgut interacts with the Malpighian tubules and forms the cryptonephridium or cryptonephridial complex^72^ (Figure 10J).

The mean volume of the L1d1 hindgut (without the pyloric cone) was 0.0156 μL with a mean area of 0.448 mm^2^ (Tables S2C and S2F). The mean volume of the L5d2 hindgut (without the pyloric cone) was 0.208 mL with a mean area of 316.2 mm^2^ (Tables S3C and S3F). Remarkably, the mean volume of the L5d6 hindgut (without the pyloric cone) was similar to L5d2 animals (0.208 mL) and had a comparable mean area of 348.2 mm^2^ (Figures 3C and 3F). Hence, the absolute hindgut volume of L5d6 and L5d2 animals was not significantly different, and no correlation or prediction with animal weight was found (Figures 4D–4D2). The volume of the hindgut as a proportion of the total larval volume was 0.7% for L1d1 (Table S2F), 15% for L5d2 (Table S3F) and 3% for L5d6 (Figure 3F).

## Discussion

In the present study, we introduce a novel μCT approach for ultra-high-resolution quantitative imaging of *M. sexta*. With this, we provide a 3D anatomic atlas that lays the anatomical base and reference for a deeper understanding of functional studies of the digestive tract of larval *M. sexta*. In addition, our approach allowed the identification of previously unknown and understudied structures, such as the crop or gastric ceca in *Manduca* larvae. Finally, it revealed the unknown, three-dimensional complexity of the larval hindgut folding pattern involved in lepidopteran fecal pellet formation.

The traditional iodine-contrasted dry scanning protocol,^42^^,^^44^^,^^68^^,^^77^ was the most suitable for complete anatomical analysis. Using this protocol, we were able to integrate the foregut (Figures 1, 2), stomodeal valve (Figures 1, 2), midgut (Figure 5), and hindgut (Figure 11) into a complete larval anatomical system. Moreover, critical point drying of midgut specimens achieved an image quality similar to histological analysis allowing the high-throughput, quantitative phenotyping of the gut epithelium in 3D (Figure 6). However, the semi-autonomous segmentation of the alimentary tract (Figure 5) was time-consuming because most tissues showed the same contrast range, and also revealed less detail than the iodixanol-contrasted hydrated scans (Figure 7). Although all tissue preservation and fixation methods cause some degree of tissue shrinkage and related artifacts, iodine contrasting seems to promote severe tissue shrinkage and the method is therefore unsuitable for volumetric quantification (Figures 3G–3J).^36^^,^^37^^,^^38^^,^^69^^,^^70^^,^^78^

Unlike the iodine-contrasted dry scanning method, the hydrated scanning method requires the larvae to ingest an artificial diet spiked or soaked with iodixanol, no fixation step is necessary. However, it is possible to use a fixative like PFA when desired. Then, minor PFA-induced shrinkage or swelling of specimens should be corrected (Table S5). Accordingly, accurate high-resolution segmentation of the alimentary tract is possible with simple thresholding. We were therefore able to quantify the mean volume and area of the alimentary tract of all three larval stages using 10 - 5 larvae per stage. This revealed that the midgut volume increases nearly by 200,000 (two hundred thousand) % over the 13–25 days between stages L1d1 and L5d6, commensurate with the increase in larval weight,^3^ highlighting the severe defoliating potential of *M. sexta*.^25^

The accurate quantification of all major gut compartments allows the calculation of locally effective midgut concentrations of applied chemicals. For instance, this type of analysis can confirm whether effective concentrations of toxins or inhibitors can be practically achieved *in vivo.*^25^ In this context, it is interesting that the volume of the midgut from an L5d6 animal (1.4 ± 0.465 mL) is comparable with the volume of the murine intestinal tract (1.34 mL).^79^ Therefore, it is reasonable to test murine concentrations of orally applied chemicals in *M. sexta* and vice versa. Recently, we have shown that *Manduca* can be used as a high-throughput platform in preclinical hypothesis testing modeling gut inflammation and host-pathogen interactions.^30^ Here, comparable dosing of applied substances will allow a faster translation from *Manduca* to murine models in line with the 3R Principle.

We have found a positive correlation between animal weight and foregut/midgut volume but not hindgut volume in animals of the developmental stages L5d2 and L5d6 (Figures 4B–4D). This finding emphasizes the cuticle-lined inextensible structure of the hindgut, which cannot grow between the molts. However, the foregut is also cuticle lined and expected to be inextensible. Here, we have shown the opposite: For the first time, we have described a crop in the foregut of *M. sexta* (Figure 7G and 7H)*.* The crop was only apparent in L5d2 animals, which may explain the absence of characterization of this structure in earlier anatomical studies,^9^ highlighting the advantages of our imaging procedure. The positive correlation between animal weight and midgut was expected because the midgut grows between molts. We calculated a simple linear regression and could predict the midgut volume from animal weight (R^2^ = 0.9437, F(1,18) = 301.5, p = <0.0001, here are the three developmental stages L1d1, L5d2, and L5d6 included) (Figure S17). This can be useful for studies working with other developmental stages allowing the estimation of midgut volume based on animal weight for custom experiments.

The iodixanol-contrasted hydrated scans also revealed the detailed structural complexity of the *M. sexta* alimentary tract. Previously, the lepidopteran digestive tract was described as “… *a simple, straight tube as long as the entire body …*”.^72^ This statement is certainly not true for *M. sexta*. In addition to the crop, we observed six major protrusions in the most anterior part of the midgut^9^ (Figures 7 and 8), resembling the gastric cecae found in *Spodoptera frugiperda* and *Heliothis virescens*.^80^ This is intriguing because lepidopterans are reported to lack gastric cecae.^81^ Of interest, gastric cecae are often associated with robust bacterial communities in insects, a topic that is now debated following research focusing on *M. sexta*.^82^^,^^83^ Accordingly, it would be interesting to determine the extent to which bacterial communities are present in the gastric ceca of *M. sexta*. The hexagonal profile, beginning with the gastric cecae, continues through the entire intestine. The midgut folding pattern is constrained by six major longitudinal muscle bundles, which form the hexagonal contours of the midgut lumen (Figures 7 and 8). This profile persists throughout the hindgut and forms six ileochambers with six corresponding ileogrooves (Figure 10). Finally, the hexagonal pattern continues through the colon and reaches the anterior rectum with six rectal grooves and the corresponding rectal lobes (Figure 10). In *M. sexta*, the fecal pallet forms in the ileum and is pressed through the colon by peristaltic contractions of the strong hindgut musculature, which gives the fecal pellets their typical hexagonal fecal grooves (Figures 11G–11K, Videos S7 and S8).^14^^,^^84^ Video S7 shows how the folding pattern of the colon imprints on the fecal pellet during this process (Figure 11). It is tempting to speculate that the increase in surface area of the fecal pellets as they acquire the hexagonal profile during passage through the colon helps restore water in the segmented anterior rectum via the cryptonephridial complex.^72^^,^^85^ However, further experiments are required to understand the reason for the segmentation of pellets, which is observed in most lepidopteran larvae.^72^ A potential application of this finding is the use of lepidopteran fecal pallet morphology as a proxy for the hindgut anatomy in a comparative evolutionary context.

One disadvantage of the iodixanol-contrasted hydrated scans is that only the contrasted structure becomes visible. The iodixanol and iodine contrasting agents are therefore complementary, and both should be adopted as standard μCT imaging methods for the analysis of insect anatomy and quantitative 3D histological phenotyping.

Finally, we examined the midgut epithelium. The lepidopteran midgut is the primary site of digestion and absorption, but another important function is ion regulation. In the clade Ditrysia, columnar cells secrete digestive enzymes and absorb nutrients^72^^,^^73^ whereas goblet cells actively transport K^+^ from the hemolymph into the gut lumen via a K^+^/2H^+^ antiporter which maintains alkaline conditions in the midgut.^72^^,^^73^^,^^86^ The columnar cells use this potassium gradient to import amino acids via an apical K^+^ symporter.^87^ Other important cell types in the gut epithelium include the enteroendocrine cells, which secrete hormones that regulate the function of the gut, and stem cells, which continuously replaceold cells.^72^^,^^73^ Cellular differentiation and regeneration in the midgut are similar in insects and mammals, reflecting conserved structures such as crypts derived from an epithelial monolayer covered with microvilli, as well as homologous Notch, K-Ras/Ras, JNK, and Wnt/wg signaling pathways^29^^,^^73^^,^^88^

We used PTA-contrasted hydrated scans of the midgut epithelium to reveal the midgut folding pattern and structure of the gut wall (Figures 6E–6G, S9, and S10). A strong second-order folding pattern was previously observed in the anterior and posterior, but only weak folding was reported in the middle midgut epithelium.^76^ This study confirms this finding (Figure S9C). In addition, we observed strong undulations in the folding of the transversal anterior midgut and posterior midgut (Figure S9). Consistent with the previous pattern, the undulations gradually declined from the anterior to the middle midgut and increased toward the posterior midgut. Moreover, PTA-contrasted hydrated scans allowed the segmentation of the ectoperitrophic space, which is (to our knowledge) the first time that the 3D visualization and quantification of the ectoperitrophic space has been reported in an insect (Figure 9).

In insects, the peritrophic matrix divides the midgut into the endoperitrophic and ectoperitrophic spaces.^72^ The peritrophic matrix (also known as the peritrophic envelope or membrane) is a random chitinous network embedded in a mucus-like envelope.^89^ Like the mucosal layer in mammals, its function is to protect the midgut epithelia from pathogens and abrasive food particles,^72^^,^^73^^,^^89^^,^^90^^,^^91^ plant allelochemicals,^92^^,^^93^ as well as facilitating the canalization of retrograde midgut currents.^94^^,^^95^ The peritrophic matrix serves as a molecular sieve and has a pore size of 24–36 nm in most lepidopterans.^73^ It is permeable to inorganic ions, sugars, amino acids, and small proteins, but impermeable to lipids, large proteins, and polysaccharides.^73^^,^^91^

Importantly, the PTA-contrasted hydrated scans agreed with *in vivo* μMRI measurements of the maximum midgut wall thickness, confirming the accurate quantification of the ectoperitrophic space (Figure 3F). However, the peritrophic matrix in most lepidopteran species is 1–10 μm thick^72^^,^^89^^,^^91^ and high-resolution *ex vivo* PTA hydrated scans could only image this indirectly (Figure 9 and Video S5). A major disadvantage of PTA as a contrast agent is its inability to penetrate the insect cuticle.^70^ Others recommend perforating the cuticle for that reason,^70^ but we found that the direct staining of the *ex vivo* midgut epithelium was satisfactory.

Taken together, our results suggest that a combination of contrast methods is required to access the complete anatomy or phenotype of the larval lepidopteran gut. If transferred to other larvae, this method will expand our knowledge about the structural evolution of the lepidopteran gut. In addition, the 3D atlas will act as a handbook for the analysis of microscopic sections or other imaging modalities covering the larval anatomy of the *M. sexta* alimentary tract. Finally, the quantitative analysis of the midgut volume will allow the calculation of locally effective midgut concentrations of applied toxins and inhibitors (Figures 4 and S17).

## Limitations of study

This study is based on 54 micro-CT scans using 49 animals representing the three most used developmental stages of *M. sexta* (L1d1, L5d2, and L5d6). Although we could predict the volume of the different gut parts from weight, in most cases, conclusions for developmental stages not represented in this study should be drawn with caution.

## STAR★Methods

### Key resources table

**Table:** 

REAGENT or RESOURCE	SOURCE	IDENTIFIER
**Chemicals, peptides, and recombinant proteins**		

Iodixanol (Visipaque)	GE Healthcare	Cat# 1133992
Hexamethyldisilazane	Sigma-Aldrich	Cat# 440191
Ethyl acetate	Biofrom	Cat# A44a
Phosphotungstic acid hydrate (PTA)	Sigma-Aldrich	Cat# P4006
Diatrizoate (Gastrografin)	Bayer	H/28/2842

**Experimental models: Organisms/strains**		

*Manduca sexta* (Lepidoptera; Sphingidae) (Linnaeus, 1763) L1d1, L5d2 and L5d6	Universität Kassel, Germany	Prof. Dr. Monika Stengl

**Software and algorithms**		

NRecon v1.7.3.0	Bruker	https://www.bruker.com
DataViewer v16.0.0	Bruker	https://www.bruker.com
CTVOX v3.3.1	Bruker	https://www.bruker.com
CTAn v1.20.8.0	Bruker	https://www.bruker.com
Amira 3D 2022.	Thermo Fisher Scientific	https://www.thermofisher.com
Final Cut Pro X v376482	Apple	https://www.apple.com
GraphPad Prism 9.2.0	Insight Partners	https://www.graphpad.com

### Resource availability

#### Lead contact

Information on resources and reagents should be directed to the lead contact, Prof. Dr. Andreas Vilcinskas (Andreas.vilcinskas@agrar.uni-giessen.de).

#### Materials availability

Any additional information required to reanalyze the data reported in this paper is available from the lead contact upon request (Andreas.vilcinskas@agrar.uni-giessen.de).

### Experimental model and subject details

For all experiments, *Manduca sexta* (Lepidoptera; Sphingidae) was grown from egg at the University of Kassel, Germany. The larvae were raised on a modified diet^2^ in an insect incubator at 24 °C and 40% relative humidity, under long-day conditions (17-h photoperiod). In the case of *M. sexta* larvae, the sex cannot be determined beyond doubt. Therefore, we cannot make any statement about the sex ratio of the animals used in this study. Working with insects does not require ethical approval in Europe or the USA.

### Method details

#### Preparation for μCT imaging

As contrast media, we tested 1% iodine solution (iodine-contrasted dry scan, whole-mount) to show the general anatomy^96^ and oral iodixanol (iodixanol-contrasted hydrated scan, whole-mount) or injected diatrizoate (diatrizoate-contrasted hydrated scan, whole-mount) to show the detailed anatomy of the alimentary tract. To visualize dissected gut components, we used PTA (PTA-contrasted hydrated scan, isolated gut) or 1% iodine solution (iodine-contrasted dry scan, isolated gut).

For the iodine-contrasted dry scan (whole-mount), L1d1, L5d2 and L5d6 specimens were anesthetized on ice for 30 min and then placed in cold 70% ethanol at ≤ 4 °C overnight. The larvae were then immersed in 1% iodine in 70% ethanol for 5 days followed by progressive dehydration in 80%, 90% and 100% ethanol. Finally, the specimens were briefly submerged in hexamethyldisilazane (Sigma-Aldrich, St. Louis, MO, USA) and air-dried under a fume hood for 20–40 days. For the iodixanol-contrasted hydrated scan (whole-mount), L1d1, L5d2 and L5d6 specimens were fed with iodixanol (Visipaque 320; GE Healthcare, Solingen, Germany) *ad libitum* for 12 h followed by euthanization with ethyl acetate (Biofrom, Nürnberg, Germany) in a killing jar. Half of the animals were scanned without fixation (L1d1 and L5d2); the other half were scanned after 12–36 h fixation in 4% PFA. The scans of the PFA fixed specimens were corrected for shrinkage (Table S5). For the diatrizoate-contrasted hydrated scan (whole-mount), larvae were cooled on ice for 30 min and injected with 0.1 mL diatrizoate (Bayer, Leverkusen, Germany) and euthanized with ethyl acetate as above.

To analyze the isolated midgut and hindgut, dissected tissue was (a) immersed in 70% ethanol overnight before staining with 1% PTA (Sigma-Aldrich) in phosphate-buffered saline (PBS) for 16 h (PTA-contrasted hydrated scan, isolated gut) and then wrapped in Parafilm (Sigma-Aldrich) to prevent drying during the scanning procedure, or (b) immersed in 1% iodine in 70% ethanol for 5 days followed by critical point drying in a Balzers CPD 030 instrument (BalTec, Pfäffikon, Switzerland).

#### μCT scans

53 specimens were scanned in a high-energy SkyScan 1173 μCT device (Bruker, Kontich, Belgium) with varying scanning parameters. Detailed information for each scan and the corresponding scanning parameters are given in Table S4.

#### NanoCT scans

In Addition, to the 53 μCt scans, one specimen was scanned in a Skyscan 2011 NanoCT (Bruker, Kontich, Belgium). The scanning parameters are given in Table S4.

#### μMRI scans

In addition to the μCt and the nanoCT scans, three *M. sexta* specimens (L5d6) were scanned in a μMRI. All MRI images were generated using a 9.4 Tesla BrukerWide Bore NMR spectrometer equipped with Mirco 2.5 gradient system and a 25-mm ^1^H quadrature coil (Bruker, Ettlingen, Germany). The insect larvae were anesthetized with 2–3 % isoflurane and placed on a home-build animal bed. For the acquisition of axial images, a rapid acquisition with relaxation enhancement (RARE) or a fast low-angle shot (FLASH) sequence was used with the following parameters: RARE,TR = 3500 ms, TE = 59.9 ms, flip angle = 90, FOV = 18 × 18 mm^2^, MS = 512 × 512 after zero filling, ST = 0.5 mm, or FLASH, TR = 200 ms, TE = 2.61 ms, flip angle = 30, FOV = 18 × 18 mm^2^, MS = 512 × 512 after zero filling, ST = 1 mm.

#### Reconstruction, preprocessing and segmentation

All datasets were reconstructed using NRecon v1.7.3.0 (Bruker, Kontich, Belgium) with a smoothing kernel of 1 or 2 before inspection using DataViewer v16.0.0 and CTVOX v3.3.1 (Bruker). The scans were preprocessed with CTAn v1.20.8.0 (Bruker, Kontich, Belgium) using a custom task list to remove the background. This list routinely executed the plug-ins Thresholding, Despeckle, Remove black Speckles or Remove Pores or ROI-Shrink-wrap, Reload, Bitwise operations, and Save bitmaps.

For segmentation, animation (Videos S1, S2, S3, S4, S5, S6, S7, and S8), illustration, and further analysis, the preprocessed datasets were processed in Amira 3D 2022.1. (Thermo Fisher Scientific, Waltham, MA, USA). The segmentation editor was used in manual mode with the freehand lasso tool or semiautomatic mode with the freehand lasso tool plus the autotrace, threshold, blow, and brush tools. For the virtual endoscopy, a surface view was generated in Amira (Figure 6). Area and volume measurements were also captured using dedicated tools in Amira. The volume and area of the endoperitrophic space were estimated by manually segmenting 1 mm of the endoperitrophic space and extrapolating the resulting values over the entire length of the midgut without the pyloric cone. Videos S1, S2, S3, S4, S5, S6, S7, and S8 were created using the animation director in Amira, and were labeled using Final Cut Pro X v376482 (Apple, Cupertino, CA, USA).

#### Microphotographs

Microscopic images of *M. sexta* were captured using a VHX-5000 digital microscope (Keyence, Osaka, Japan) with a standard zoom lens (VH-Z20R) and a free-angle observation system (VHX-S550E). Larvae were cooled on ice for 30 min and fixed to a Petri dish with Leukosilk tape (BSN Medical, Hamburg, Germany) for imaging.

### Quantification and statistical analysis

For Statistical analysis, we used GraphPad Prism 9.2.0 (Insight Partners, New York City). First, we calculated absolute and relative mean values for area and volume quantifications. To compensate for volume or shrinkage artifacts due to PFA fixation (Scan # 11-19), we compared the animal length before fixation and after imaging (Table S5). Then, we corrected the area and volume calculation in the proportion of the length difference. In addition, we compared two micro-CT protocols (*ex vivo* PTA vs. ex vivo iodine) by comparison of maximal midgut epithelial thickness, the maximal midgut fold thickness measurements and tissue shrinkage, employing unpaired t-tests. Next, we compared the maximal midgut wall thickness measurements of the two protocols (*ex vivo* PTA vs. ex vivo iodine). We validated these measurements with *in vivo* micro-MRI measurements using a one-way ANOVA. Then, we calculated Pearson correlations and simple linear regression models of the animal weights and the volume of different gut parts. Finally, we compared the relative and absolute volumes of the different gut parts between the developmental stages L5d2 to L5d6 using unpaired t tests. We used parametric tests for normally distributed data and nonparametric tests when the data did not satisfy normal distribution. The presented bar charts represent the mean and the standard deviation. In all figures, every data point represents a single animal. Scatterplots show the 95 confidence intervals (dashed lines) and the trend line (lines). Boxplots show the 25th–75th percentiles, whiskers show the min-max data with all data points, and the center represents the median. The following significance levels have been used: ns = P > 0.05, ∗ = P≤0.05, ∗∗P≤0.01, ∗∗∗ = P≤0.001 and ∗∗∗∗ = P≤0.0001. Statistical details of each experiment can be found in the respective figure legend.

## Data Availability

• Data is available from the lead contact Prof. Dr. Andreas Vilcinskas (Andreas.vilcinskas@agrar.uni-giessen.de). • This study did not create code. • Any additional information required to reanalyze the data reported in this work is available from the lead contact upon reasonable request.
