# Supplementary material for: A quantitative micro-tomographic gut atlas of the lepidopteran model insect Manduca sexta
Source: iScience. 2023 May 5;26(6):106801. doi: 10.1016/j.isci.2023.106801 (PMC10291339; doi:10.1016/j.isci.2023.106801)
Supplement: Document S1. Figures S1–S17 and Tables S1–S5 [file mmc1.pdf]

iScience, Volume *26*

Supplemental information

A quantitative micro-tomographic gut atlas of the lepidopteran model insect *Manduca sexta*

Anton G. Windfelder, Jessica Steinbart, Ulrich Flögel, Jan Scherberich, Marian Kampschulte, Gabriele A. Krombach, and Andreas Vilcinskas

Table S1: Abbreviations and nomenclature for the anatomical structures, related to Fig. 1-11.

Anatomical System	Abbreviation	Anatomical structure
General anatomy		
	**1L**	First leg
	**2L**	Second leg
	**3L**	Third leg
	**A1–A8**	Abdominal segments 1–8
	**AB**	Abdomen
	**TAS**	Terminal abdominal segment
	**DD**	Dorsal diaphragm
	**DOV**	Dorsal vessel
	**FB**	Fat body
	**HE**	Hemocoel
	**MESO**	Mesothorax (second thoracic segment)
	**META**	Metathorax (third thoracic segment)
	**MP**	Malpighian tubules
	**PL1–PL4**	Prolegs 1–4
	**PRO**	Prothorax (first thoracic segment)
	**SA**	Seta
	**SG**	Silk gland
	**TH**	Terminal horn
	**TPL**	Terminal proleg
	**TX**	Thorax
	**VD**	Ventral diaphragm
Head and mouthparts		
	**A**	Antenna
	**CL**	Clypeus
	**H**	Head
	**LB**	Labium
	**LBR**	Labrum
	**MA**	Mandible
	**MX**	Maxilla
	**OC**	Oral cavity (functional mouth)
	**PO**	Preoral cavity
	**ST**	Stemma (simple eye)
	**TN**	Tentorium
Tracheal system		
	**AT**	Atrium
	**SIP**	Sieve plate
	**SP**	Spiraculum
	**T**	Trachea
	**V**	Valve
Muscular system		
	**DC**	Dorsocoxal muscle
	**DL**	Dorsolongitudinal muscle
	**DP**	Dorsopleural muscle
	**DV**	Dorsoventral muscle
	**LM**	Longitudinal visceral musculature (gut)
	**M**	Visceral muscle (gut)
	**MAD**	Mandibular adductor
	**ML**	Muscular layer of the foregut
	**SC**	Spiracular muscle
	**VL**	Ventrolongitudinal muscle
Nervous system		
	**FG**	Frontal ganglion
	**MG**	Mesothoracic ganglion
	**PG**	Prothoracic ganglion
	**SEG**	Subesophageal ganglion
	**SPG**	Supraesophageal ganglion or brain
	**VNC**	Ventral nerve cord
Digestive system		
	**CG**	Coleogroove
	**GC**	Gut content
	**PC**	Pyloric cone

Table S2: Mean area, volume or proportional volume of different *Manduca sexta* L1d1 gut parts, related to Fig. 3. (**A**–**F**) Mean (n = 5) area, mean volume and proportional volume based on iodixanol-contrasted hydrated scans of whole-mount larvae.

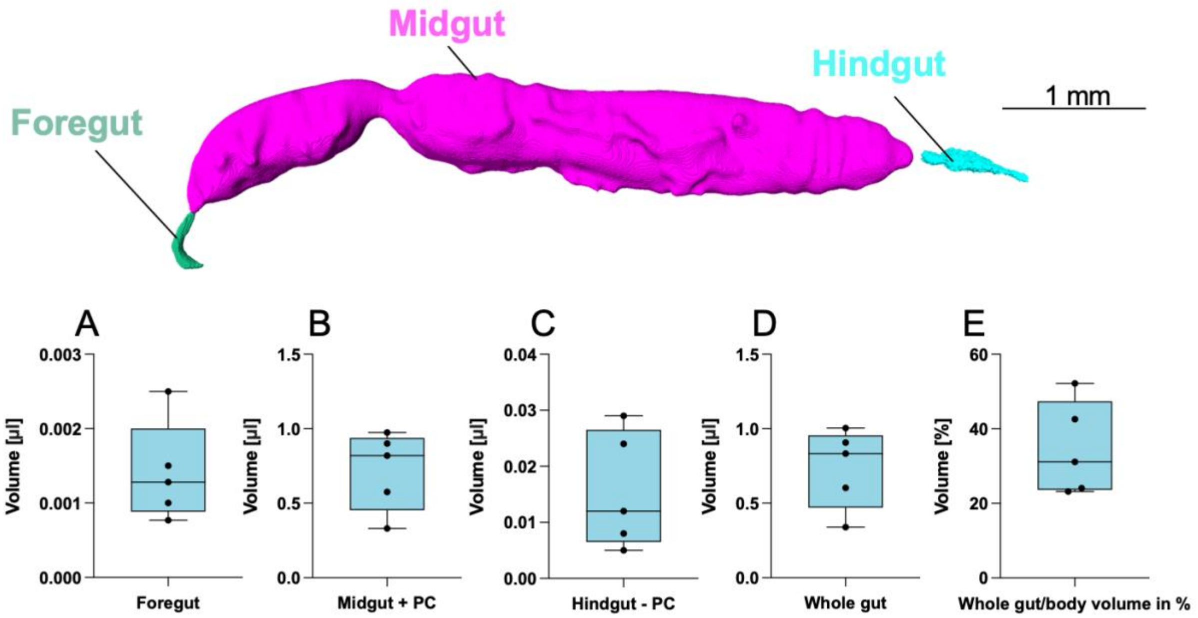

F

L1d1	Mean area [mm^2]	Mean volume [µl]	Mean label/whole body volume in %
Foregut (n =5)	0.124 ± 0.06	0.0014 ± 0.0006	0.064 ± 0.02074
Midgut + PC* (n = 5)	7.466 ± 2.038	0.72 ± 0.2645	33.82 ± 12.33
Hindgut -PC* (n = 5)	0.448 ± 0.2249	0.0156 ± 0.01041	0.736 ± 0.4996
Whole gut (n = 5)	8.036 ± 1.998	0.737 ± 0.2674	34.62 ± 12.51
Whole body (n = 5)	27.31 ± 6.045	2.147 ± 0.5194	100.00

+: with, - without, PC: pyloric cone, ±: Standard deviation

Table S3: Mean area, volume or proportional volume of different *Manduca sexta* L5d2 gut parts related to Fig. 3. (**A**–**F**) Mean (n = 5) area, mean volume and proportional volume based on iodixanol-contrasted hydrated scans of whole-mount larvae.

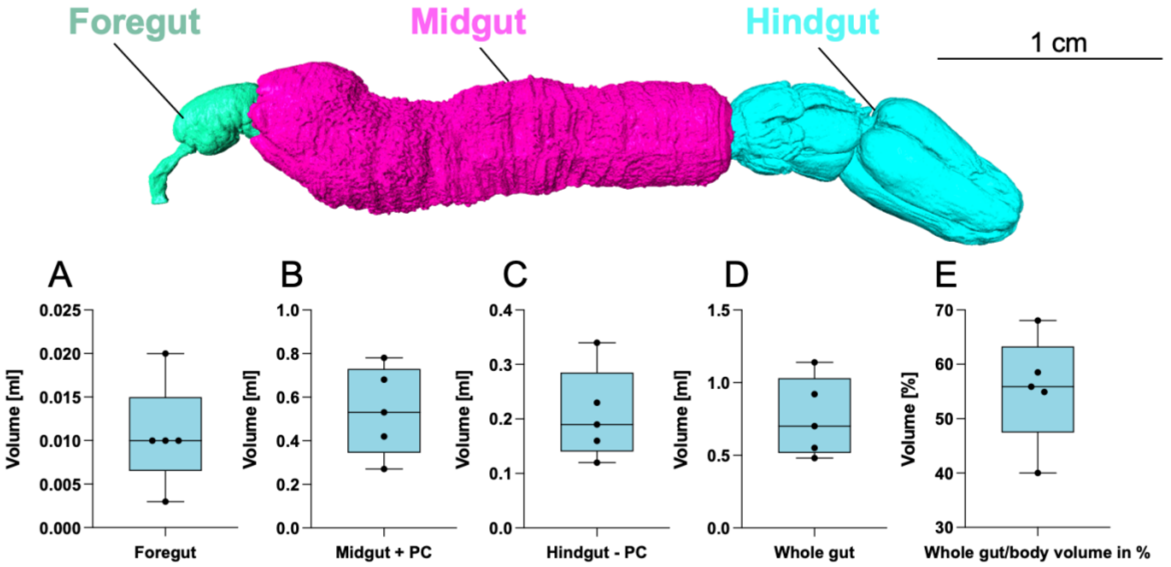

F

L5d2	Mean area [mm²]	Mean volume [ml]	Mean label/whole body volume in %
Foregut (n =5)	36.8 ±12.58	0.0106 ±0.006066	0.934 ±0.5231
Midgut + PC* (n = 5)	722.8 ±210.4	0.536 ±0.2028	39.37 ±9.595
Hindgut -PC* (n = 5)	316.2 ±89.97	0.208 ±0.08408	15.17 ±3.236
Whole gut (n = 5)	1076 ±251.8	0.758 ±0.2721	55.47 ±10.09
Whole body (n = 5)	2554 ±593.7	1.346 ±0.3097	100.00

+: with, - without, PC: pyloric cone, ±: Standard deviation

Table S4: Detailed information for each scan and the corresponding scanning parameters, related to STAR Methods (µCT scans). AD* = air dried; CPD**: critical point dried.

#	Figure	Animal or specimen	Scan modality	Contrast agent	Connected scans	Source voltage [kV]	Source current [µA]	Image pixel size [µm]	Filter [mm]	Rotation step [˚]	Frame averaging	Scan duration [h:m:s]
1	1-2	L5d6	dry scan AD*	iodine	0	60	133	6.7	0.5 Al	0.2	4	01:42:57
2	3-4, 9	L5d6	dry scan AD*	iodine	2	60	133	19.7	0.5 Al	0.3	4	01:10:37
3	Table 1	L5d6	hydrated scan	iodixanol (oral)	3	40	200	19.1	no	0.3	2	00:41:07
4	S9	L5d6	hydrated scan	diatrizoate	3	70	110	19.9	0.5 Al	0.3	4	01:04:17
5	4H-J	L5d6	dry scan CPD**	iodine	1	45	80	5.3	0.5 Al	0.17	8	04:55:03
6	4H-J	L5d6	dry scan CPD**	iodine	1	40	115	5.3	no	0.17	6	03:39:08
7	4H-J	L5d6	dry scan CPD**	iodine	1	40	115	5.3	no	0.17	6	03:26:50
8	/	L5d6	dry scan CPD**	iodine	1	40	115	4.9	no	0.18	6	03:27:27
9	/	L5d6	dry scan CPD**	iodine	3	40	115	5.3	no	0.17	6	03:42:20
10	9G-K	fecal pellet	native	native	1	40	95	5.4	no	0.25	4	01:47:31.
11	4E-G	L5d6	hydrated scan	PTA	1	60	133	10.5	0.5 Al	0.3	4	01:10:01
12	4E-G	L5d6	hydrated scan	PTA	1	60	133	10.5	0.5 Al	0.3	4	01:05:00
13	Tab. 1	L5d6	hydrated scan	iodixanol (oral)	3	50	160	19.9	no	0.3	2	00:38:27
14	Tab. 1	L5d6	hydrated scan	iodixanol (oral)	3	50	160	19.6	no	0.3	2	00:42:20
15	Tab. 1	L5d6	hydrated scan	iodixanol (oral)	3	50	160	19.9	no	0.3	2	00:38:19
16	Tab. 1	L5d6	hydrated scan	iodixanol (oral)	3	50	160	19.9	no	0.3	2	00:38:56
17	Tab. 1	L5d6	hydrated scan	iodixanol (oral)	3	50	160	19.9	no	0.3	2	00:48:45
18	Tab. 1	L5d6	hydrated scan	iodixanol (oral)	3	50	160	19.9	no	0.3	2	00:48:38
19	Tab. 1	L5d6	hydrated scan	iodixanol (oral)	3	50	160	19.9	no	0.3	2	00:48:26
20	Tab. 1	L5d6	hydrated scan	iodixanol (oral)	3	50	160	19.9	no	0.3	2	00:48:23
21	Tab. 1	L5d6	hydrated scan	iodixanol (oral)	3	50	160	19.9	no	0.3	2	00:40:45
22.1	4 + S4	L5d6	hydrated scan	PTA	3	50	160	7.47	0.5 Al	0.25	4	01:44:34
22.2	4 + S4	L5d6	hydrated scan	PTA	5	50	160	7.47	0.5 Al	0.25	4	02:33:15
23	4 + S4	L5d6	hydrated scan	PTA	7	50	160	7.47	0.5 Al	0.25	4	01:44:00
24	4 + S4	L5d6	hydrated scan	PTA	7	50	160	7.47	0.5 Al	0.25	4	01:45:04
25	4 + S4	L5d6	hydrated scan	PTA	7	50	160	7.47	0.5 Al	0.25	4	01:57:32
26	4 + S4	L5d6	hydrated scan	PTA	7	50	160	7.47	0.5 Al	0.25	4	02:45:01
27	4 + S4	L5d6	hydrated scan	PTA	6	50	160	7.47	0.5 Al	0.25	4	03:42:03
28	4 + S4	L5d6	hydrated scan	PTA	6	50	160	7.47	0.5 Al	0.25	4	02:03:53
29	4 + S4	L5d6	hydrated scan	PTA	6	50	160	7.47	0.5 Al	0.25	4	03:40:06
30	4 + S4	L5d6	hydrated scan	PTA	6	50	160	7.47	0.5 Al	0.25	4	01:54:22
31	4 + S4	L5d6	hydrated scan	PTA	6	50	160	7.47	0.5 Al	0.25	4	01:45:31

Table S4 (continue): Detailed information for each scan and the corresponding scanning parameters. AD* = air dried; CPD**: critical point dried.

#	Figure	Animal or specimen	Scan modality	Contrast agent	Connected scans	Source voltage [kV]	Source current [µA]	Image pixel size [µm]	Filter [mm]	Rotation step [˚]	Frame averaging	Scan duration [h:m:s]
32	/	L5d2	dry scan AD*	iodine	3	60	133	8.9	0.5 Al	0.3	4	01:28:14
33	/	L5d2	dry scan AD*	iodine	5	60	133	6.0	0.5 Al	0.3	4	01:19:49
34	/	L5d6	dry scan AD*	iodine	3	60	133	19.8	0.5 Al	0.3	4	01:22:11
35	/	L5d6	dry scan AD*	iodine	3	60	133	19.9	0.5 Al	0.3	4	01:27:05
36	/	L5d6	dry scan AD*	iodine	3	60	133	19.9	0.5 Al	0.3	4	01:24:55
37	/	L5d6	dry scan AD*	iodine	3	60	133	19.9	0.5 Al	0.3	4	01:18:50
38	/	L5d6	dry scan AD*	iodine	2	60	133	19.9	0.5 Al	0.3	4	01:19:07
39	/	L5d6	dry scan AD*	iodine	3	60	133	19.9	0.5 Al	0.3	4	02:13:26
40		L1d1	hydrated scan	iodixanol (oral)	1	40	200	4.98	0.5 Al	0.24	4	00:42:39
41		L1d1	hydrated scan	iodixanol (oral)	1	40	200	4.98	0.5 Al	0.24	4	00:42:37
42		L1d1	hydrated scan	iodixanol (oral)	1	40	200	4.98	0.5 Al	0.24	4	00:42:31
43		L1d1	hydrated scan	iodixanol (oral)	1	40	200	4.98	0.5 Al	0.24	4	00:44:03
44		L1d1	hydrated scan	iodixanol (oral)	1	35	180	5.69	0.5 Al	0.17	8	01:47:27
45		L5d2	hydrated scan	iodixanol (oral)	2	50	160	14.92	0.5 Al	0.3	4	01:11:25
46		L5d2	hydrated scan	iodixanol (oral)	2	50	160	13.15	0.5 Al	0.3	4	01:16:43
47		L5d2	hydrated scan	iodixanol (oral)	2	50	160	12.08	0.5 Al	0.3	4	01:28:13
48		L5d2	hydrated scan	iodixanol (oral)	2	50	160	14.93	0.5 Al	0.3	4	01:24:45
49		L5d2	hydrated scan	iodixanol (oral)	2	50	160	14.22	0.5 Al	0.3	4	01:23:54
50		Egg	dry scan AD*	iodine	1	40	100	4.35	no	0.18	6	02:13:09
51		L1d1	dry scan AD*	iodine	1	40	200	4.97	no	0.2	4	01:13:37
52		L1d1	dry scan AD*	iodine	1	40	100	4.6	no	0.18	6	05:15:31
53		L1d1	dry scan AD*	iodine	1	40	200	4.97	no	0.2	4	01:00:53
54		L1d1 (Head/ *NanoCT*)	dry scan AD*	iodine	1	60	140	1.50	no	0.2	6	03:12:37

Table S5: Detailed information about the animals, volume and shrinkage corrections for the oral iodixanol contrast hydrated scan (whole-mount), related to STAR Methods (Preparation for µCT imaging).

#	Animal	Length before fixation [cm]	Length after imaging [cm]	Change in length (%)
3	L5d6	No fixation	/	/
13	L5d6	7.7	7.8	+ 1.3
14	L5d6	8.4	8.1	– 2.51
15	L5d6	8.7	7.96	– 8.47
16	L5d6	7.9	8.07	+2.15
17	L5d6	7.9	8.88	– 0.27
18	L5d6	7.2	7.28	+1.11
19	L5d6	6.2	6.24	+0.65
20	L5d6	6.3	6.3	0.00
21	L5d6	6.1	6.2	+1.64
45	L5d2	No fixation	/	/
46	L5d2	No fixation	/	/
47	L5d2	No fixation	/	/
48	L5d2	No fixation	/	/
49	L5d2	No fixation	/	/
40	L1d1	No fixation	/	/
41	L1d1	No fixation	/	/
42	L1d1	No fixation	/	/
43	L1d1	No fixation	/	/
44	L1d1	No fixation	/	/

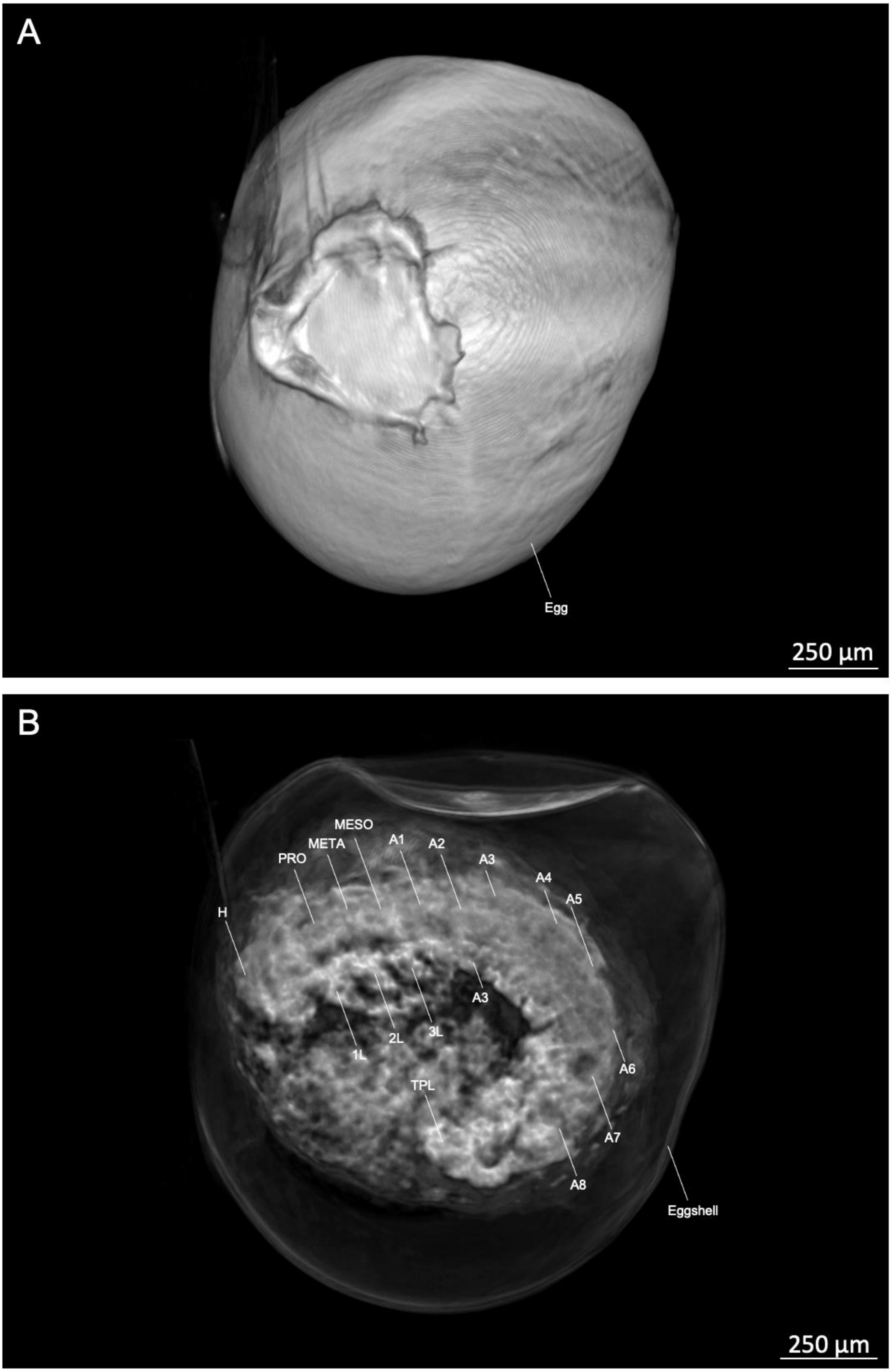

Fig. S1: Late Manduca sexta Embryo (iodine-contrasted dry scan, whole-mount) , related to Fig. 2. 1L-3L: First-three legs, A1-A8: Abdominal segments, 1–8H: Head, MESO: Mesothorax (second thoracic segment), META: Metathorax (third thoracic segment), PRO: Prothorax (first thoracic segment), TPL: Terminal proleg.

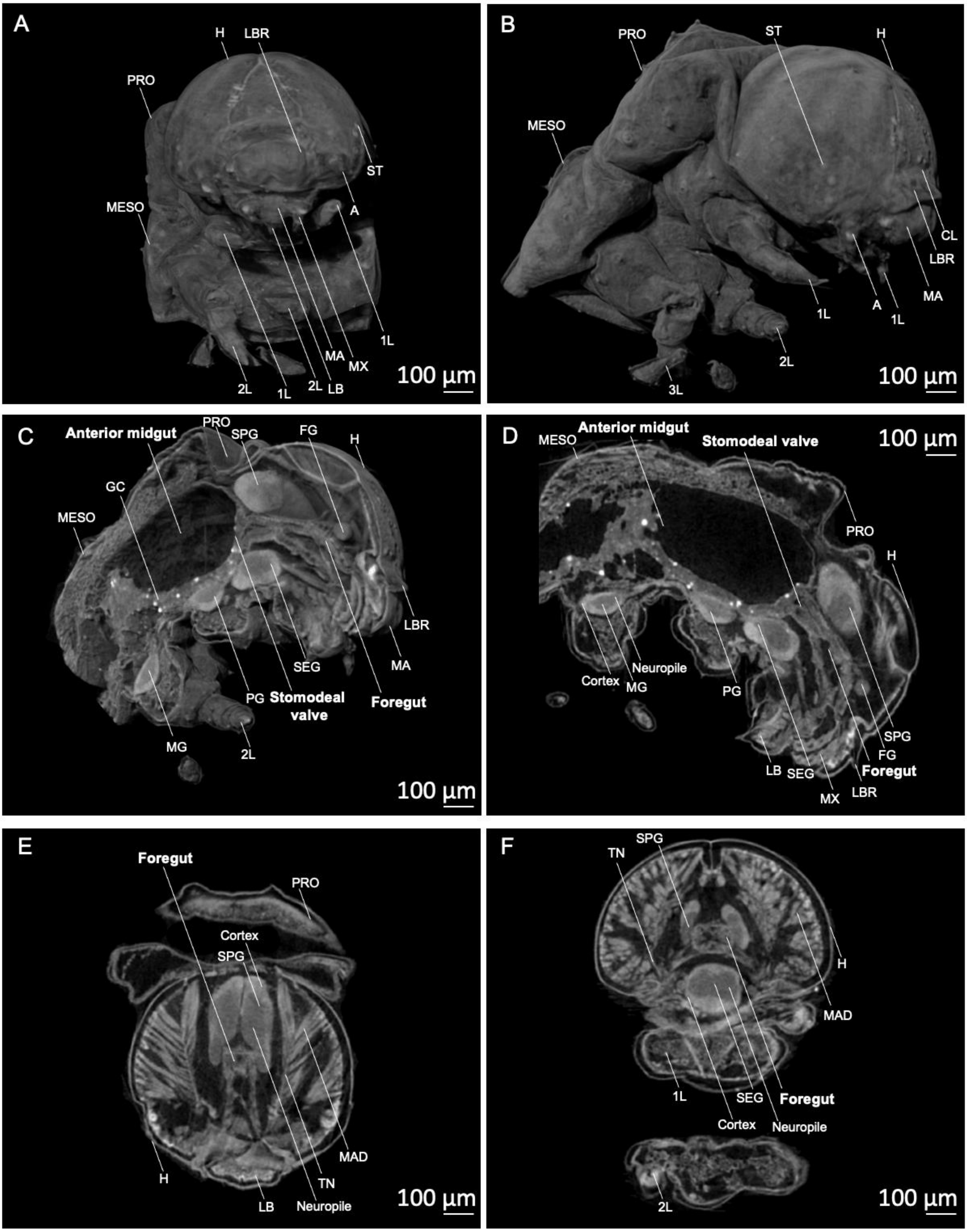

Fig. S2: Early L1D1 Larva (head and thorax, iodine-contrasted dry scan, nano CT), related to Fig. 2. 1L-3L: First-three legs, A: Antenna, A1-A8: Abdominal segments 1–8, CL: Clypeus, FG: Frontal ganglion, GC: Gut content, H: Head, LB: Labium, LBR: Labrum, MA: Mandible, MAD: Mandibular adductor, MESO: Mesothorax (second thoracic segment), META: Metathorax (third thoracic segment), MG: Mesothoracic

ganglion, MX: Maxilla, PG: Prothoracic ganglion, PRO: Prothorax (first thoracic segment), SEG: Subesophageal ganglion, SPG: Supraesophageal ganglion or brain, ST: Stemma (simple eye), TN: Tentorium, TPL: Terminal proleg.

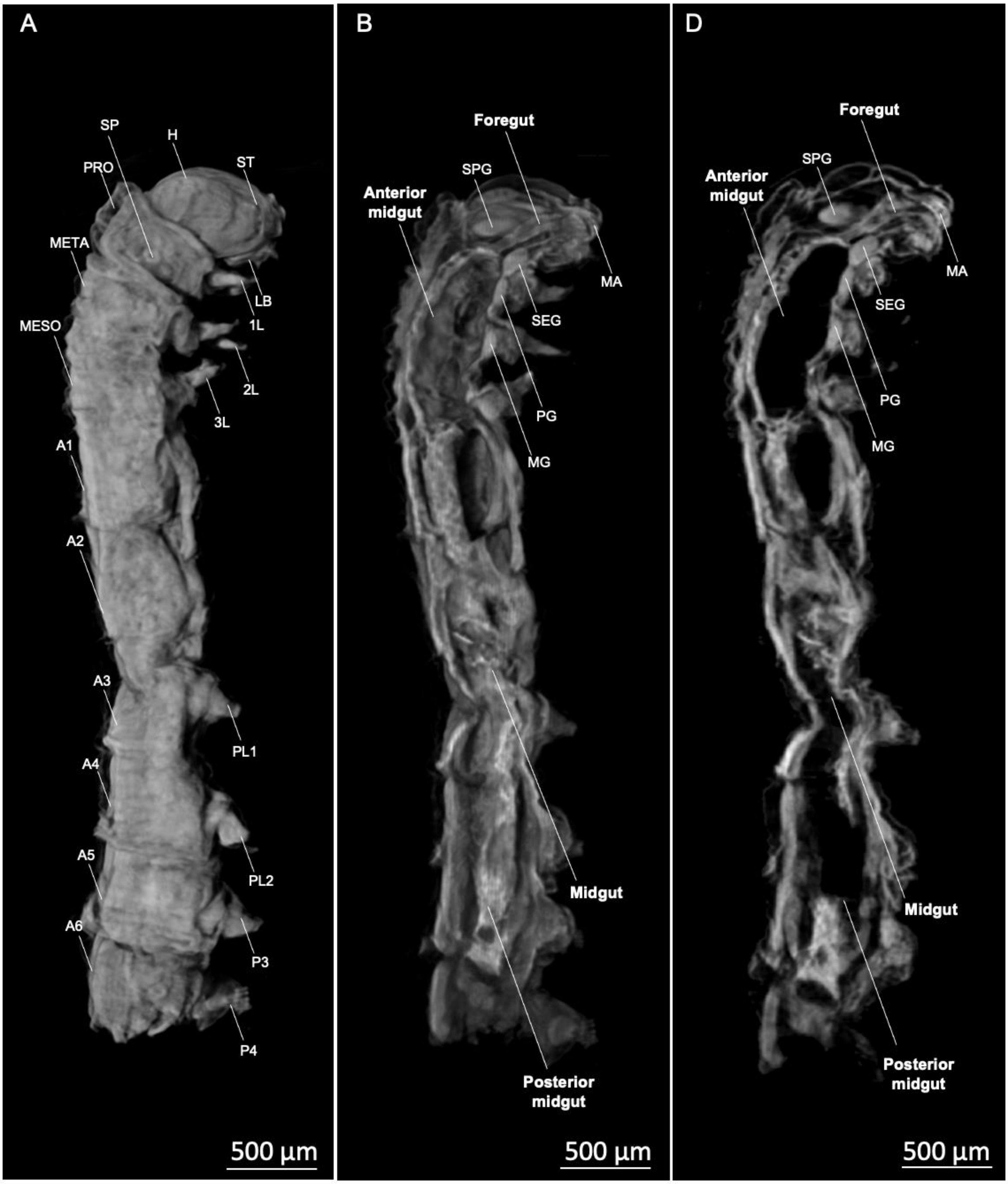

Fig. S3: Early L1D1 Larva (iodine-contrasted dry scan, whole-mount), related to Fig. 2 and 5. 1L-3L: First-three legs, A1-A8: Abdominal segments 1–8, H: Head, LB: Labium, LBR: Labrum, MA: Mandible, MESO: Mesothorax (second thoracic segment), META: Metathorax (third thoracic segment), MG: Mesothoracic ganglion, PG: Prothoracic ganglion, PL1-4: Prolegs 1–4, PRO: Prothorax (first thoracic segment), SEG: Subesophageal ganglion SP: Spiraculum, SPG: Supraesophageal ganglion or brain, ST: Stemma (simple eye).

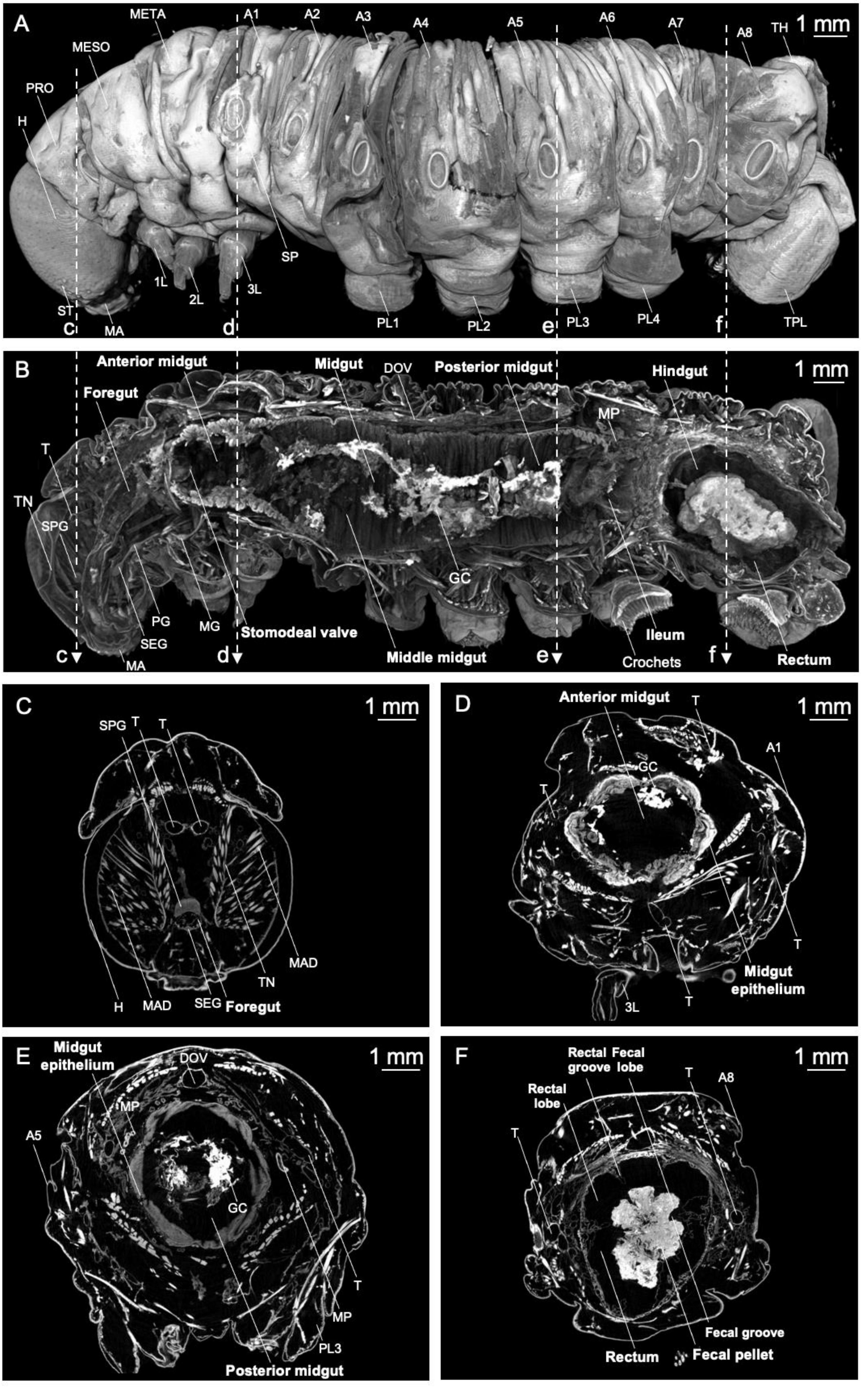

A
1 mm
H
PRO
MESO
META
A1
A2
A3
A4
A5
A6
A7
A8
TH
SP
1L
2L
3L
ST
MA
PL1
PL2
PL3
PL4
TPL
c
d
e
f
B
1 mm
Anterior midgut
Foregut
Midgut
DOV
Posterior midgut
Hindgut
MP
T
TN
SPG
PG
SEG
MA
MG
Stomodeal valve
Middle midgut
GC
Ileum
Crochets
Rectum
c
d
e
f
C
1 mm
SPG
T
T
H
MAD
SEG
Foregut
TN
MAD
D
1 mm
Anterior midgut
T
A1
GC
T
T
Midgut epithelium
T
3L
T
E
1 mm
Midgut epithelium
DOV
MP
A5
GC
T
MP
PL3
Posterior midgut
F
1 mm
Rectal groove
Fecal lobe
Rectal lobe
T
A8
T
Fecal groove
Rectum
Fecal pellet

Fig. S4: Early L5D2 larva (iodine-contrasted dry scan, whole-mount) , related to Fig. 2. L5d2 larva from lateral (**A**), sagittal (**B**), and four different axial sections (**C-F**). 1L-3L: First-three legs, GC: Gut content, H: Head, MA: Mandible, MAD: Mandibular adductor, MESO: Mesothorax (second thoracic segment), META: Metathorax (third thoracic segment), MG: Mesothoracic ganglion, MX: Maxilla, PG: Prothoracic ganglion, PRO: Prothorax (first thoracic segment), SEG: Subesophageal ganglion SPG: Supraesophageal ganglion or brain, SP: Spiraculum, ST: Stemma (simple eye), TH: Terminal horn, TN: Tentorium, TPL: Terminal proleg DOV: Dorsal vessel, MP: Malpighian tubules, T: Trachea, PL1-4: Prolegs 1–4, A1-A8: Abdominal segments 1–8.

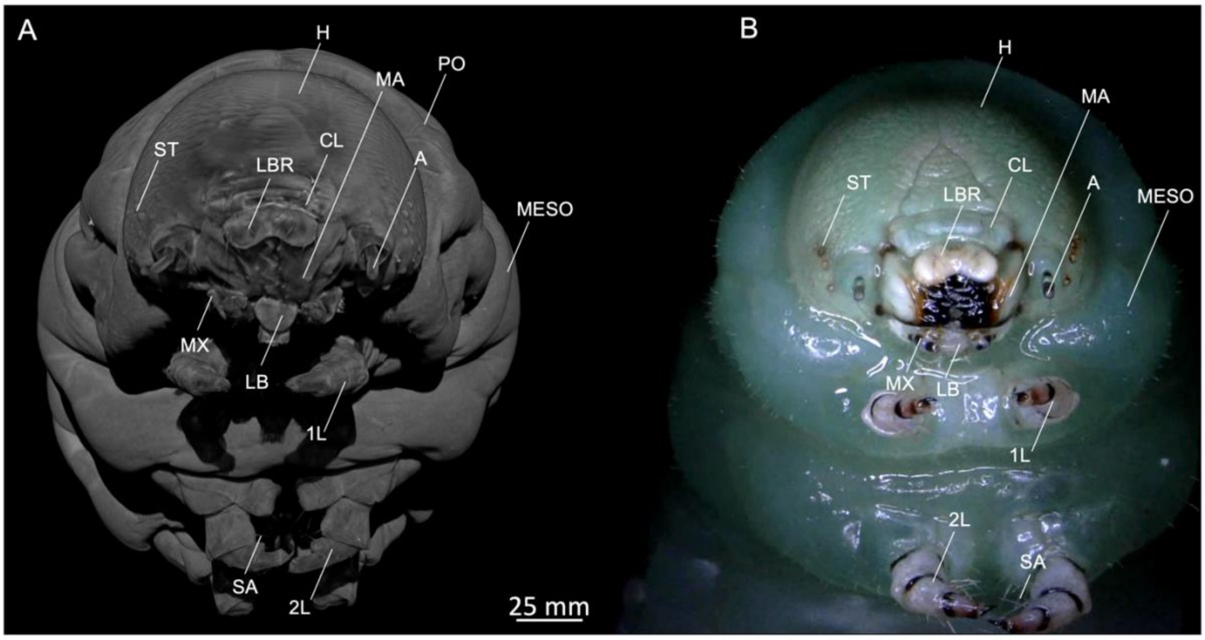

Fig. S5: Volume rendering and microphotography of the thorax and head of a late L5d6 *Manduca sexta* larva, related to Fig. 1-2. Volume rendering (A) and microphotography (B) of the thorax and head of a late L5d6 larva 1L: first leg, 2L: second leg, CL: clypeus, H: head, LBR: labrum, MA: mandible, MESO: mesothorax (second thoracic segment), MX: maxilla, PRO: prothorax (first thoracic segment), SA: seta, ST: stemma (simple eye).

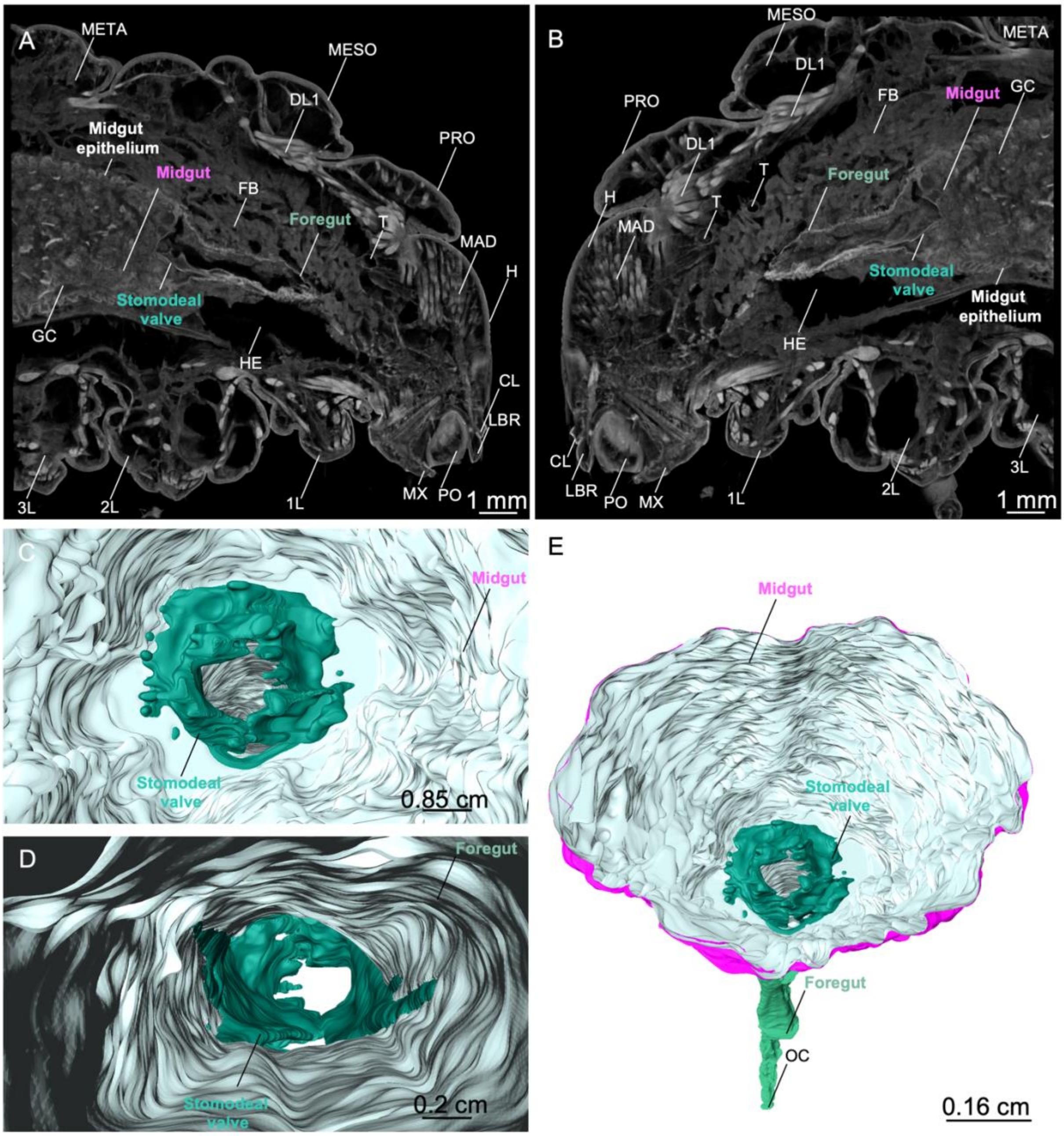

Fig. S6: Volume rendering of the thorax and head of a late L5d6 *Manduca sexta* larva contrasted with 1% iodine, related to Fig. 1-2. (**A, B**) Volume rendering of the thorax and head of a late L5d6 larva, sagittal cross-sections. 1–3L: legs 1–3, CL: clypeus, DL: dorsolongitudinal muscle, FB: fat body, GC: gut content, H: head, HE: hemocoel, LBR: labrum, MAD: mandibular adductor, MESO: mesothorax (second thoracic segment), META: metathorax (third thoracic segment), MX: maxilla, PO: preoral cavity, PRO: prothorax (first thoracic segment), T: trachea. The stomodeal valve is shown from the (**C**) posterior and (**D**) anterior perspectives. (**C**) Segmented foregut, stomodeal valve, and the anterior midgut. OC: oral cavity (functional mouth).

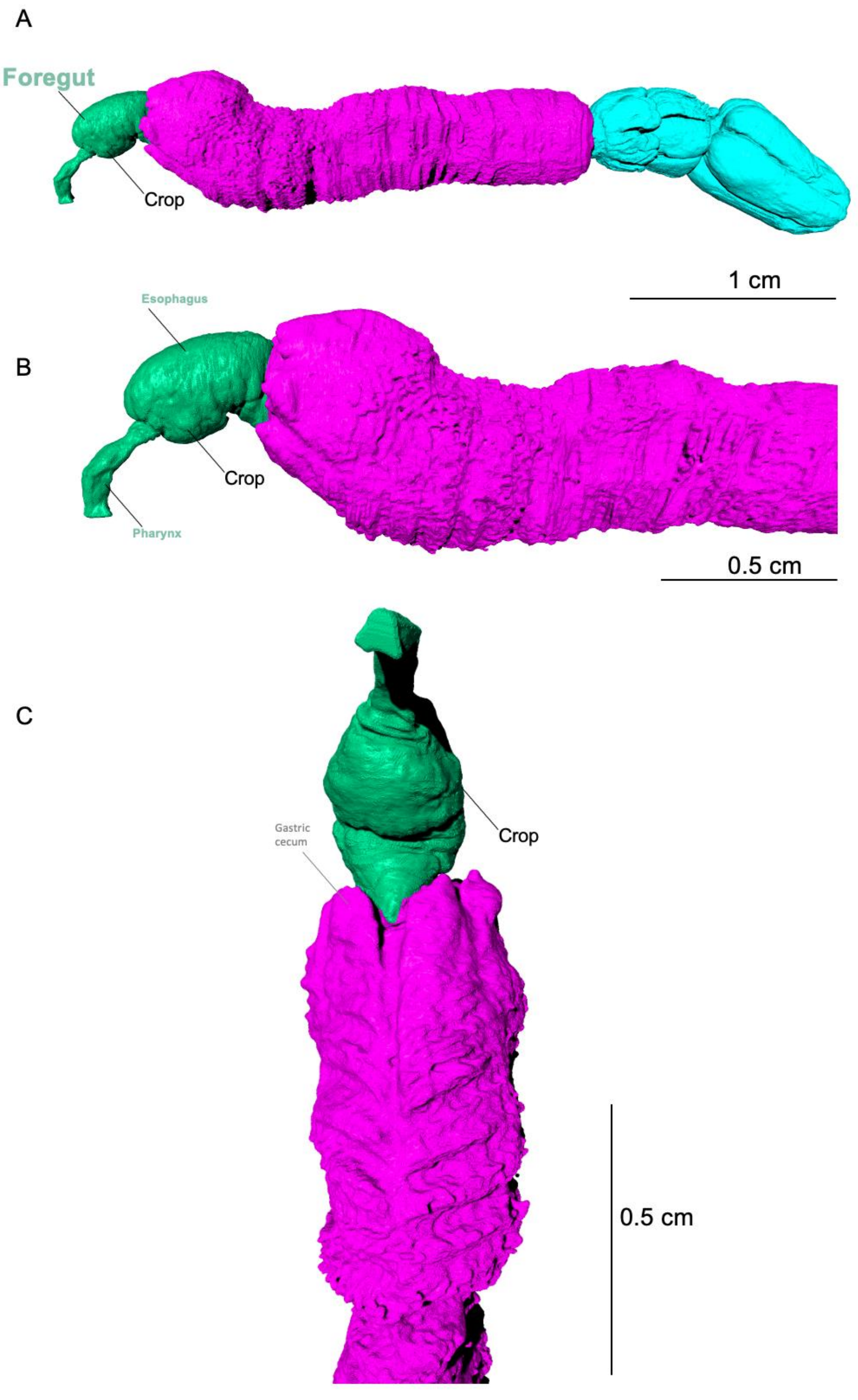

Fig. S7: Volume rendering of the gut with crop from an L5d2 animal (oral iodixanol contrasting, hydrated scan, whole-mount), related to Fig. 7.

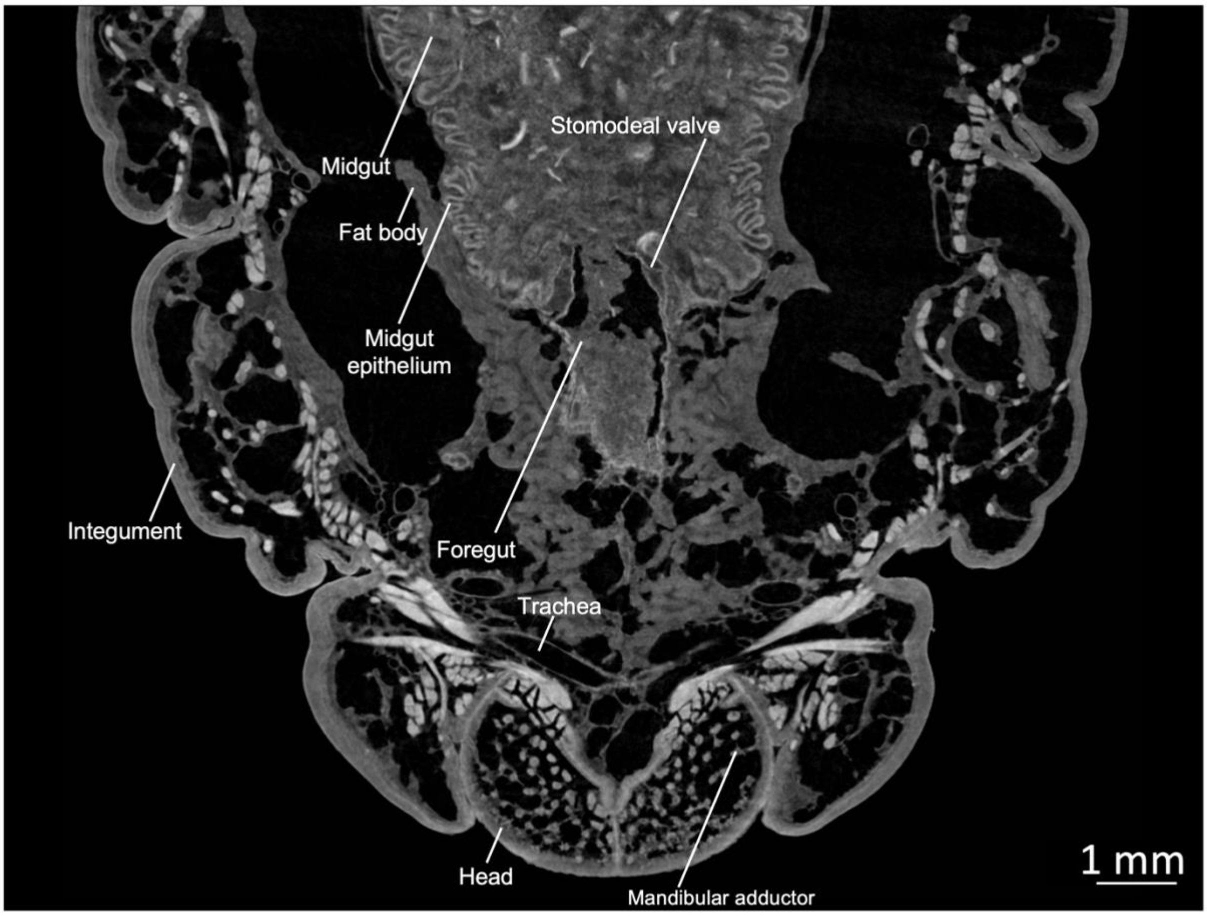

Fig. S8: Late L5d6 *Manduca sexta* larvae in the coronal plane contrasted with 1% iodine, related to Fig. 5.

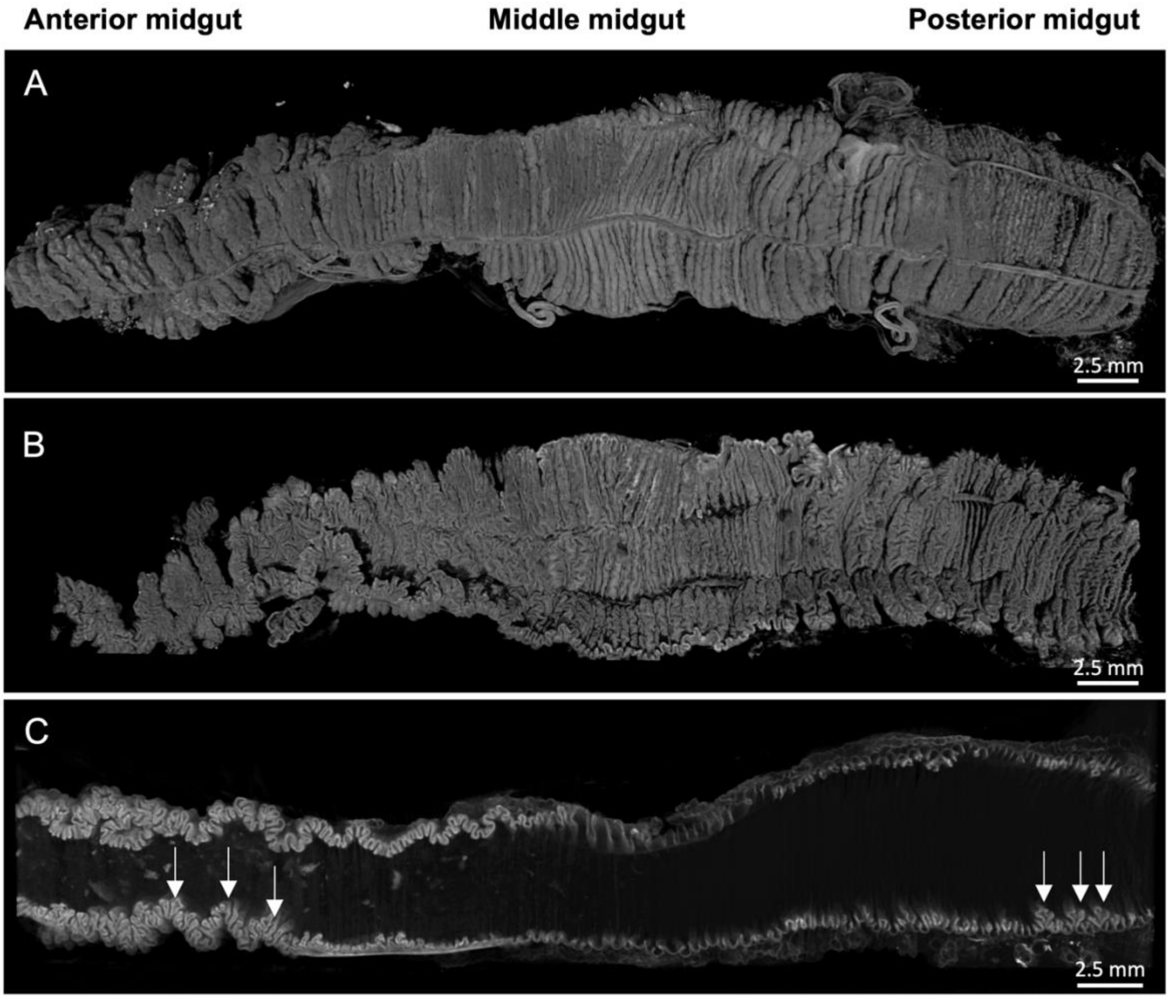

Fig. S9: Epithelial topography of the anterior, middle and posterior midgut of a late L5d6 *Manduca sexta* larva contrasted with PTA, related to Fig. 5-6. (**A**) lateral (outside) view of the 3D volume rendering or (**B**) sagittal (internal) view. **C** shows the midgut in sagittal the plane.

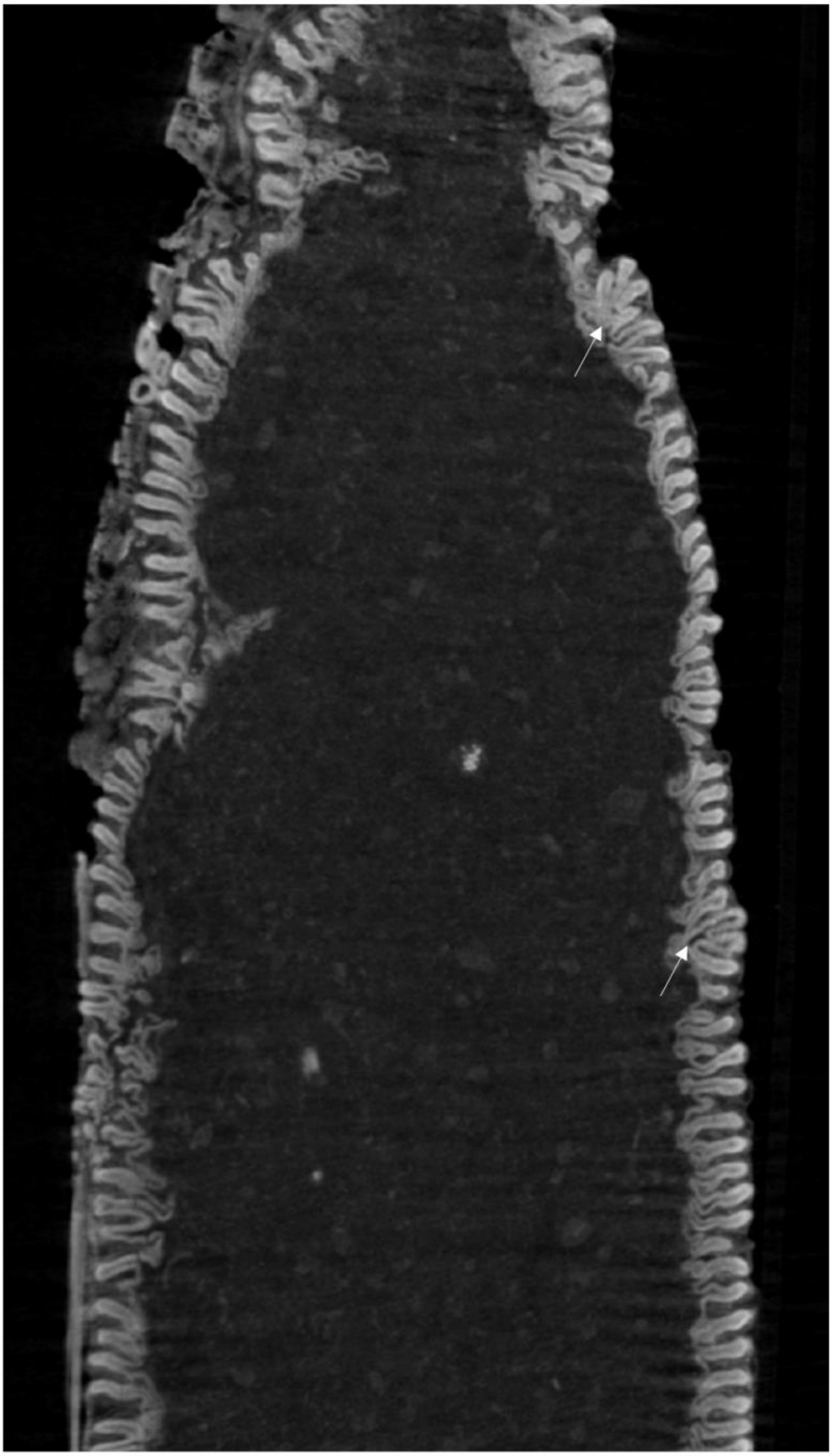

Fig. S10: Epithelial topography of the isolated anterior and middle midgut of a late L5d6 *Manduca sexta* larva contrasted with PTA, related to Fig. 5-6. Image shows the sagittal plane and arrows indicate second-order folding.

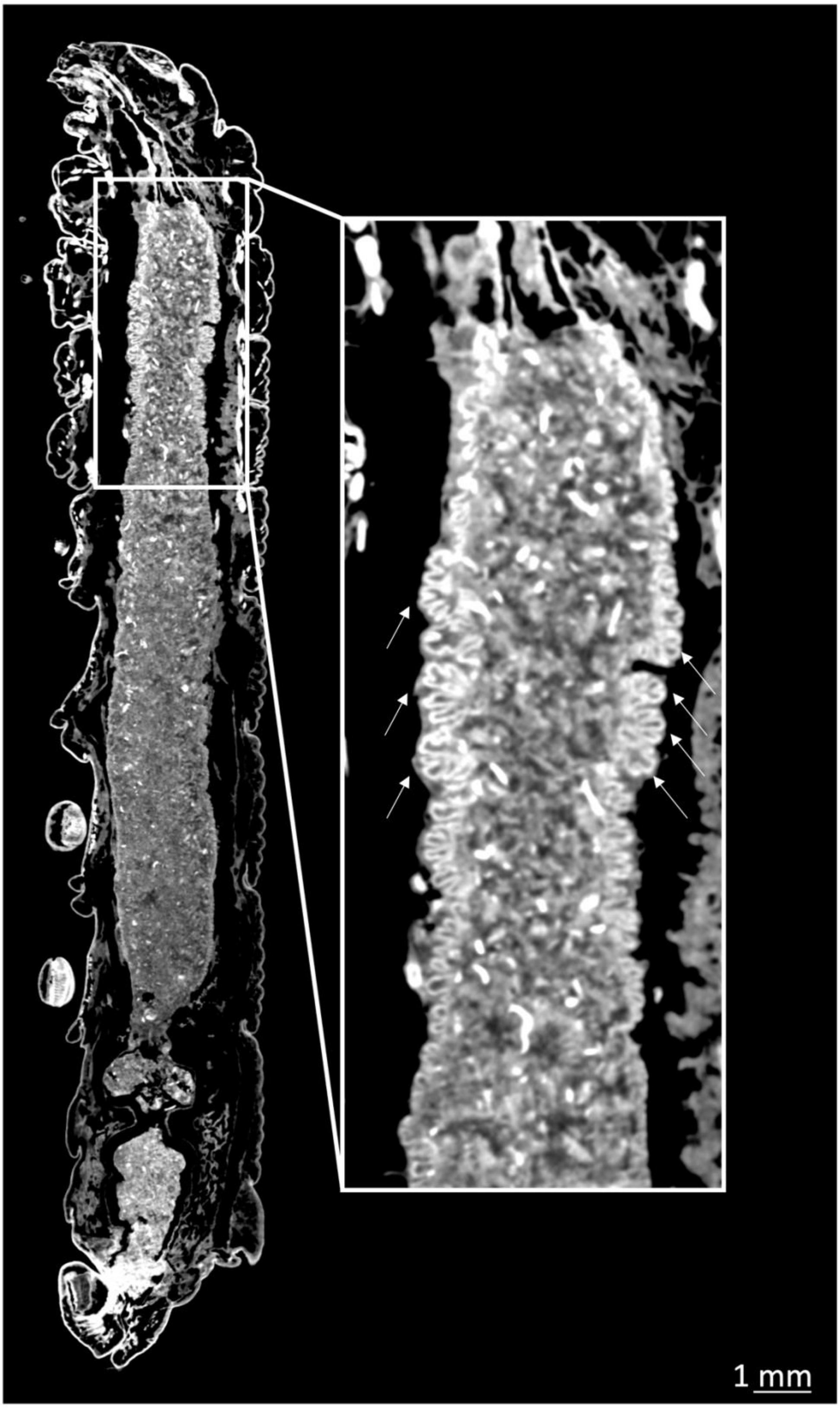

Fig. S11 Epithelial topography of the midgut of a late L5d6 *Manduca sexta* larva contrasted with iodine, focusing on the anterior and middle midgut, related to Fig. 5. Image shows the sagittal plane and arrows indicate second-order folding.

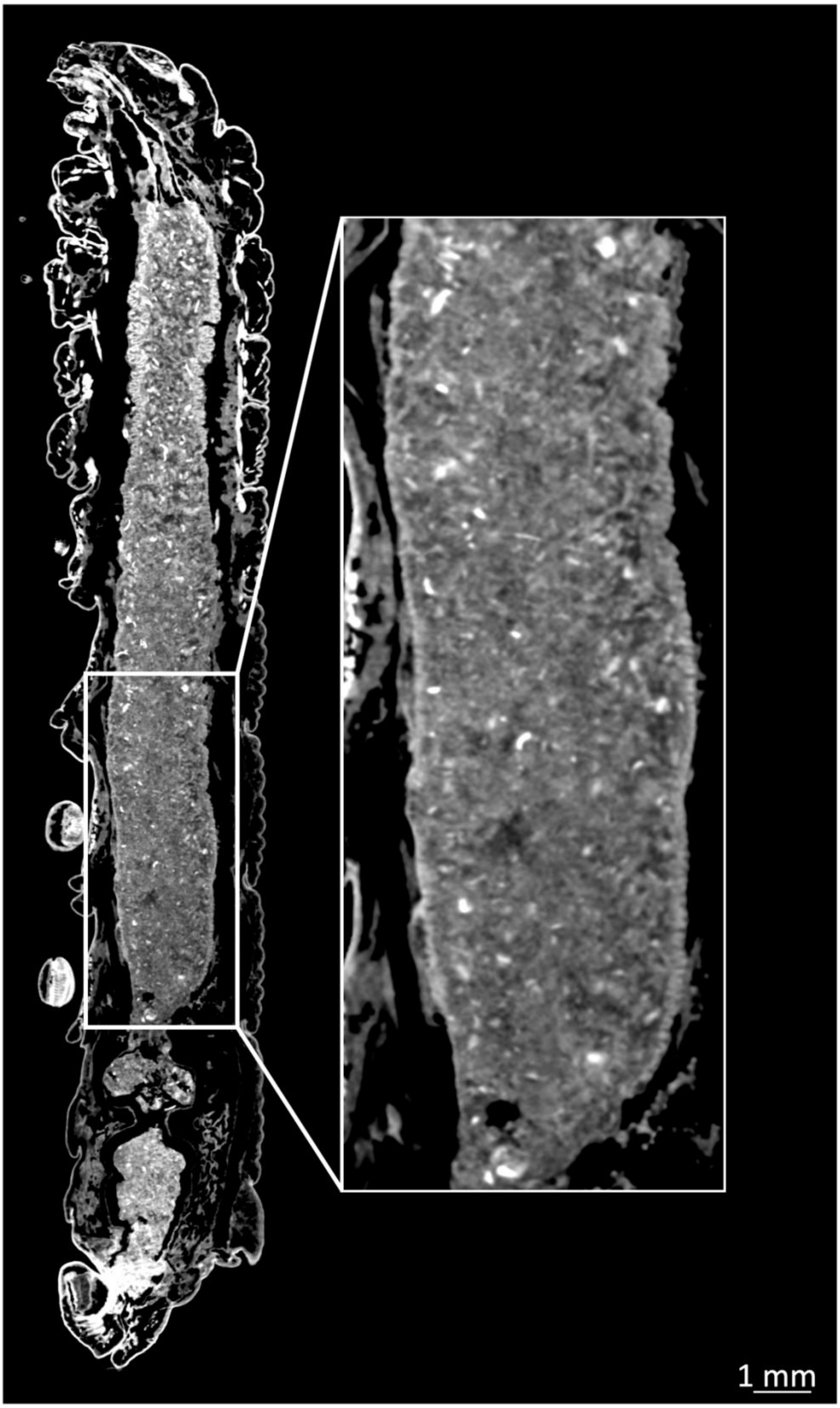

Fig. S12 Epithelial topography of the midgut of a late L5d6 *Manduca sexta* larva contrasted with iodine, focusing on the posterior midgut, related to Fig. 5. Image shows the sagittal plane and arrows indicate second-order folding.

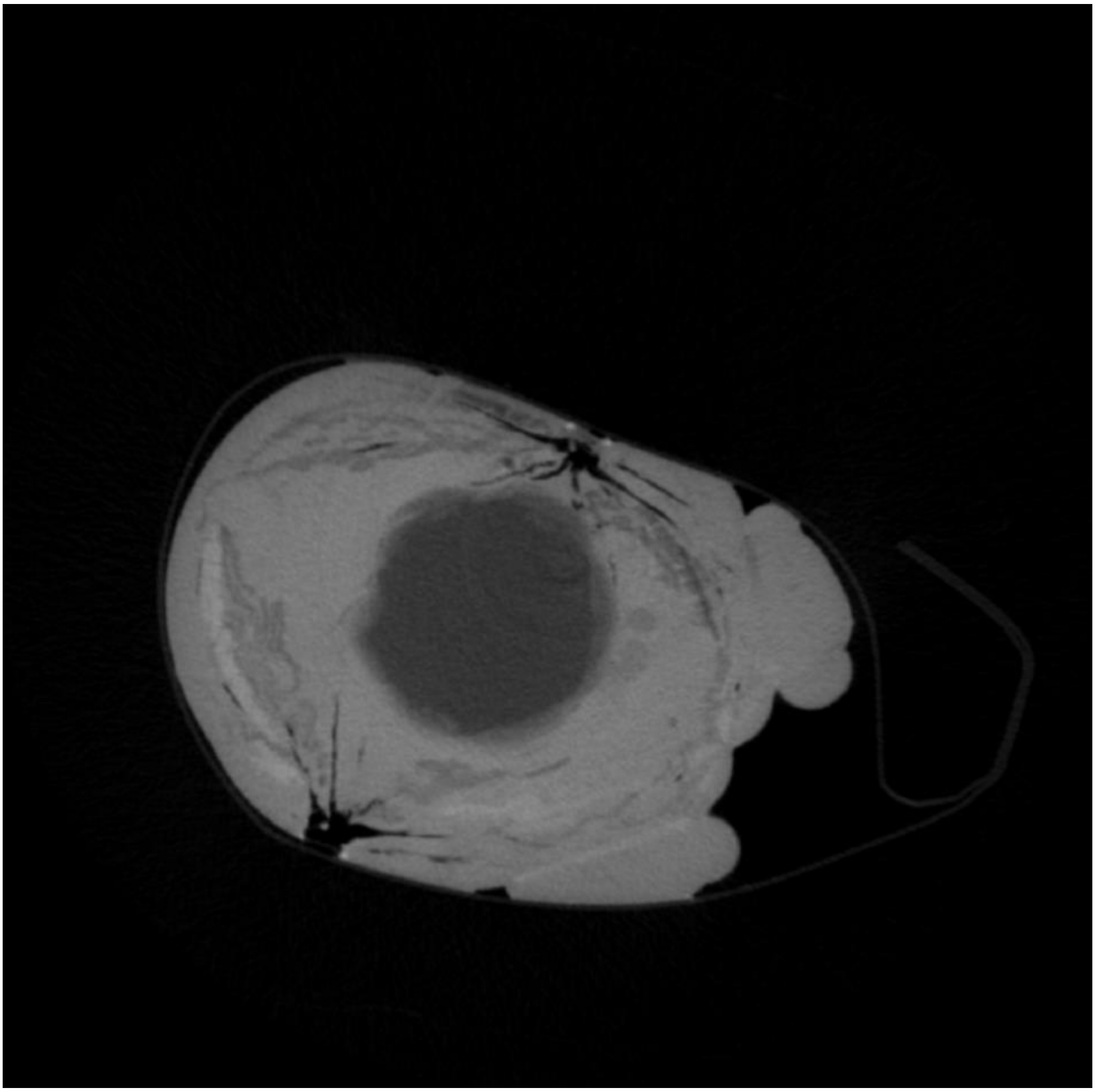

Fig. S13: Injection of diatrizoate (Gastrografin) followed by µCT scan in a late L5d6 *Manduca sexta* larva), related to STAR Methods (Preparation for µCT imaging). Axial cross-section at the level of the first prolegs. Note that the gut is blurred and poorly defined.

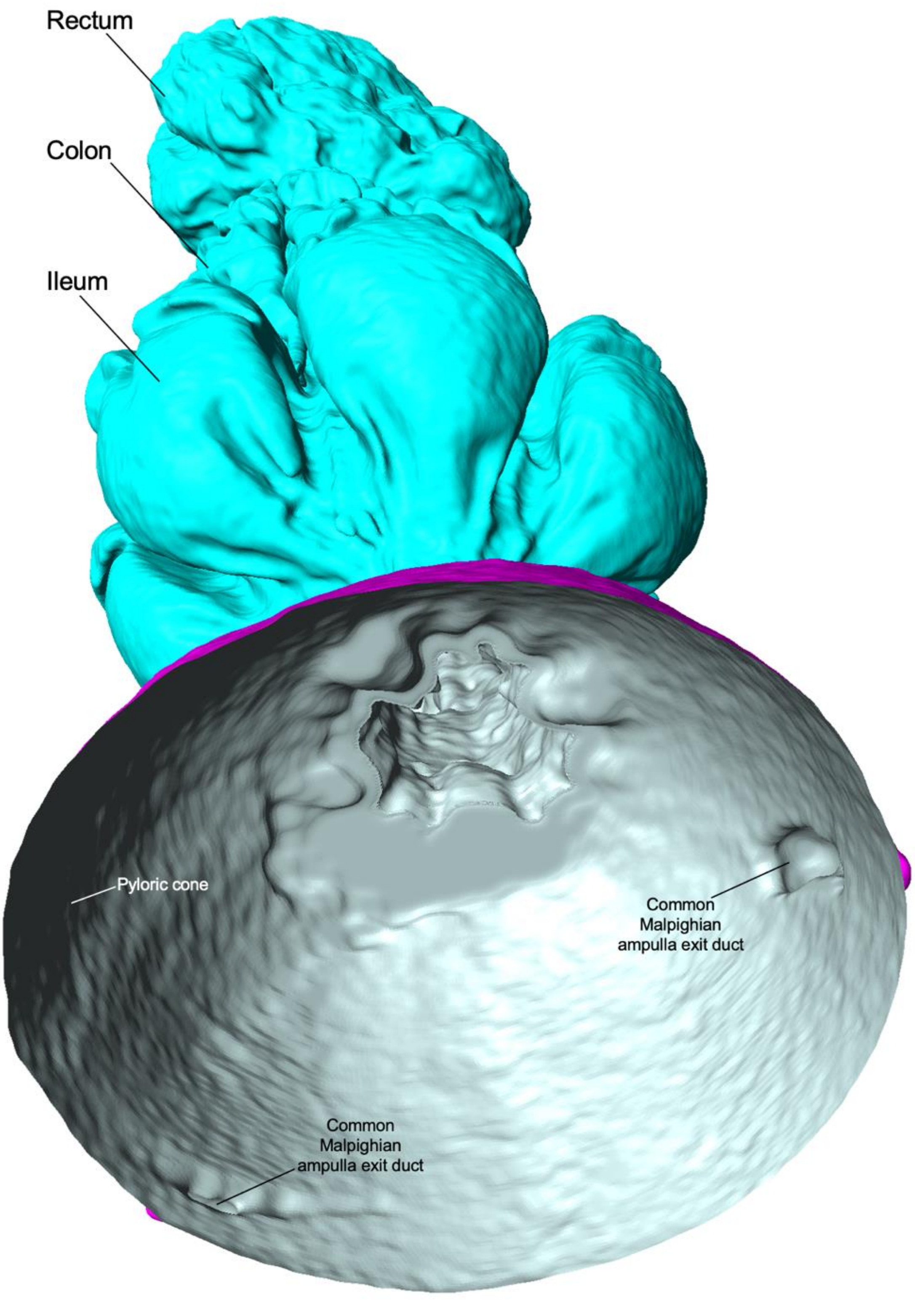

Fig. S14: Surface model of the pyloric cone with the two insertions of the common Malpighian ampulla exit ducts in a late L5d6 *Manduca sexta* larva related, to Fig. 8.

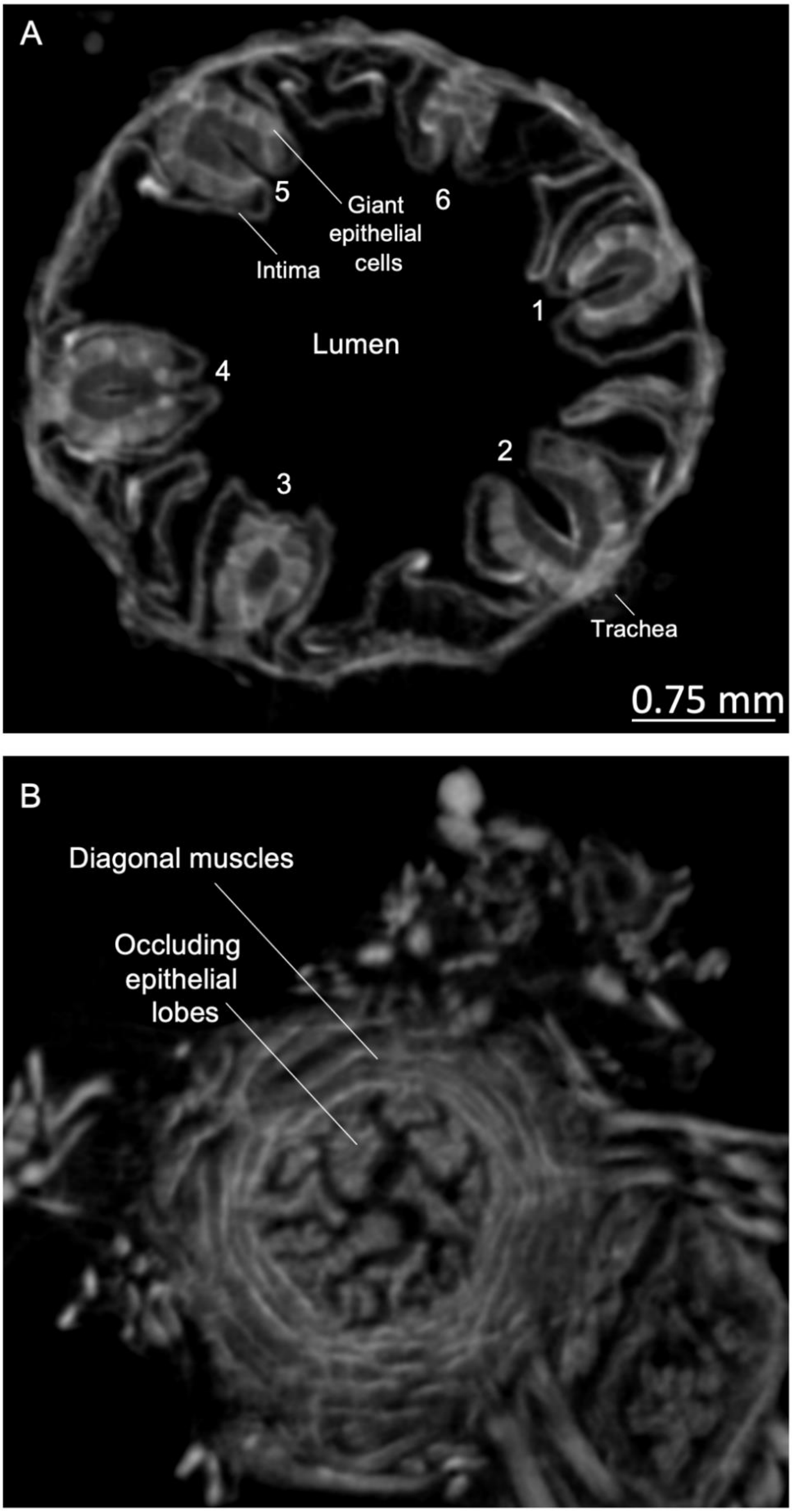

Fig. S15: Giant epithelium (giant epithelial cells, enlarged view of Fig. 8E,I) of the ileum and occluding epithelial lobes of the colon (axial plane) in a late L5d6 *Manduca sexta* larva contrasted with iodine, related to Fig. 10.

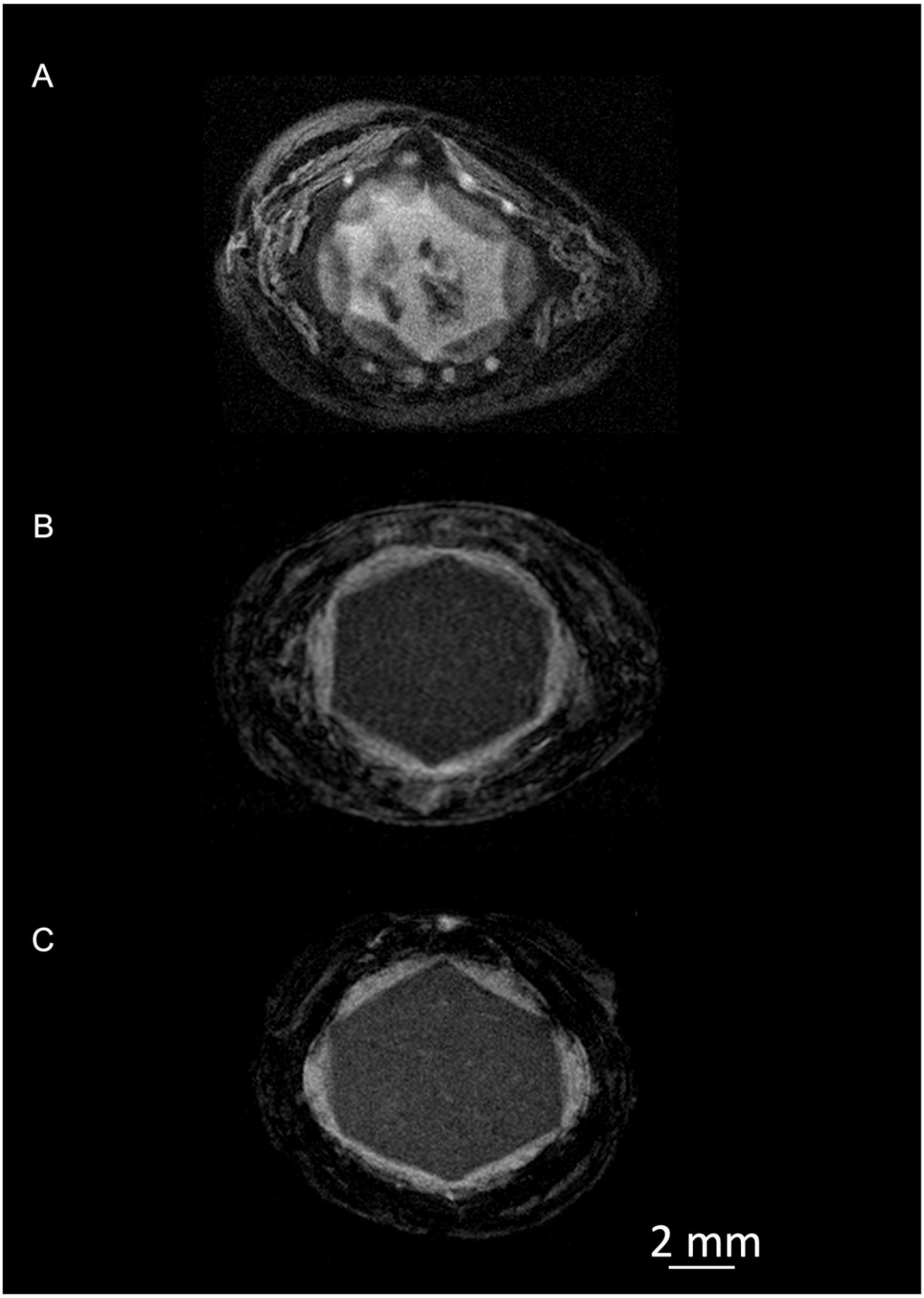

Fig. S16: Images captured by µMRI of a late L5d6 *Manduca sexta* larva (axial orientation), related to Fig. 3. (A) Rapid acquisition with relaxation enhancement **(**RARE) µMRI image. **(B,C)** fast low-angle shot **(**FLASH) µMRI images.

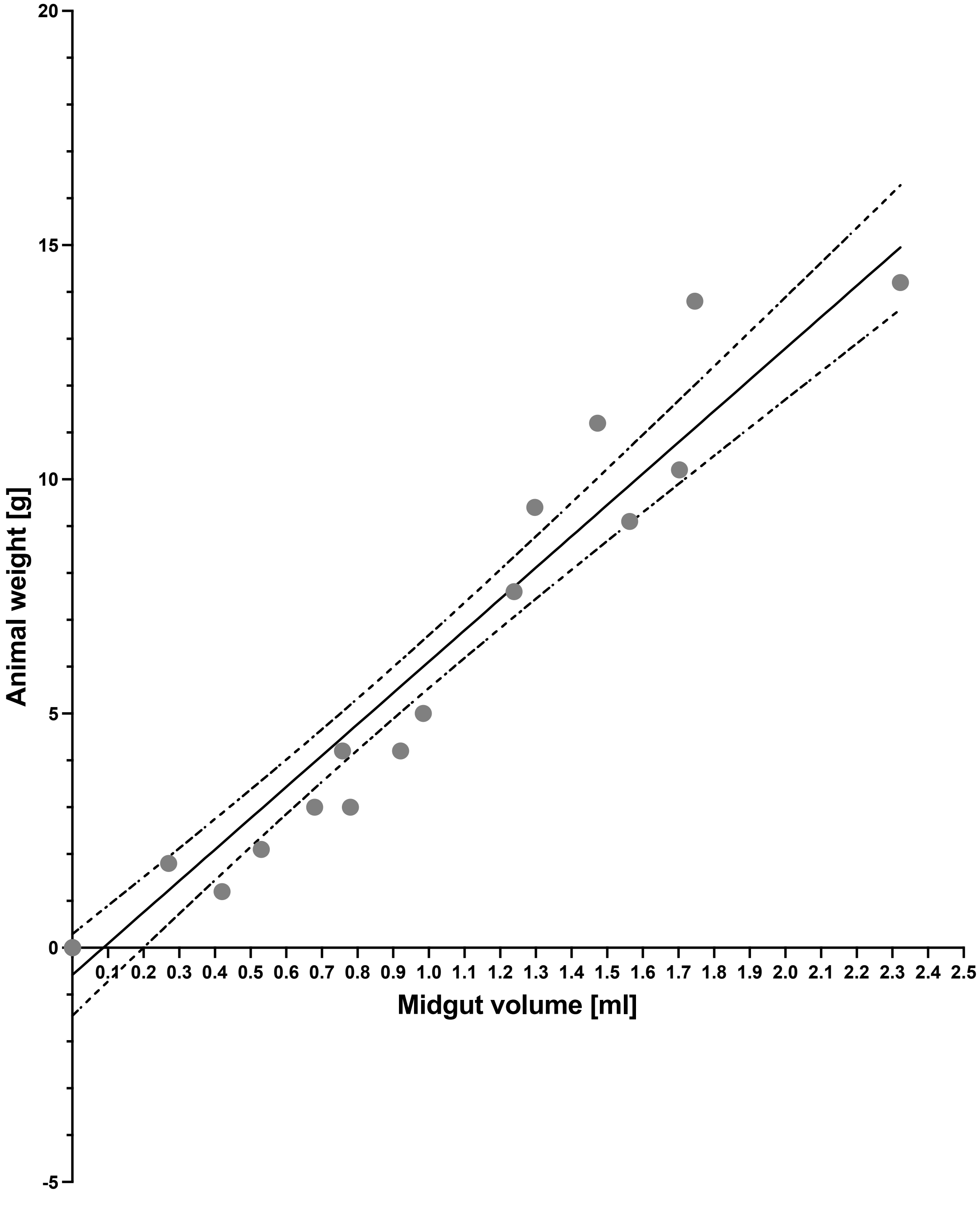

Fig. S17: Simple linear regression between animal weight and midgut volume (here with the developmental stage L1d2), related to Fig. 4.
